# BODIPY nanoparticles functionalized with lactose for cancer-targeted and fluorescence imaging-guided photodynamic therapy

**DOI:** 10.1038/s41598-022-06000-5

**Published:** 2022-02-15

**Authors:** Duy Khuong Mai, Chanwoo Kim, Joomin Lee, Temmy Pegarro Vales, Isabel Wen Badon, Koushitak De, Sung Cho, Jaesung Yang, Ho-Joong Kim

**Affiliations:** 1 Department of Chemistry, Chosun University, Gwangju, 61452 Korea; 2 Department of Chemistry, Chonnam National University, Gwangju, 61186 Korea; 3 Department of Chemistry, Yonsei University, Wonju, 26493 Gangwon Korea; 4 College of Food and Nutrition, Chosun University, Gwangju, 61452 Korea; 5 Department of Natural Sciences, Caraga State University, 8600 Butuan City, Philippines; 6 Department of Cellular and Molecular Medicine, College of Medicine, Chosun University, Gwangju, 61452 Korea

**Keywords:** Chemistry, Materials science

## Abstract

A series of four lactose-modified BODIPY photosensitizers (PSs) with different substituents (-I, -H, -OCH_3_, and -NO_2_) in the *para*-phenyl moiety attached to the *meso*-position of the BODIPY core were synthesized; the photophysical properties and photodynamic anticancer activities of these sensitizers were investigated, focusing on the electronic properties of the different substituent groups. Compared to parent BODIPY **H**, iodine substitution (BODIPY **I**) enhanced the intersystem crossing (ISC) to produce singlet oxygen (^1^O_2_) due to the heavy atom effect, and maintained a high fluorescence quantum yield (Φ_F_) of 0.45. Substitution with the electron-donating methoxy group (BODIPY **OMe)** results in a significant perturbation of occupied frontier molecular orbitals and consequently achieves higher ^1^O_2_ generation capability with a high Φ_F_ of 0.49, while substitution with the electron-withdrawing nitro group (BODIPY **NO2**) led a perturbation of unoccupied frontier molecular orbitals and induces a forbidden dark S_1_ state, which is negative for both fluorescence and ^1^O_2_ generation efficiencies. The BODIPY PSs formed water-soluble nanoparticles (NPs) functionalized with lactose as liver cancer-targeting ligands. BODIPY **I** and **OMe** NPs showed good fluorescence imaging and PDT activity against various tumor cells (HeLa and Huh-7 cells). Collectively, the BODIPY NPs demonstrated high ^1^O_2_ generation capability and Φ_F_ may create a new opportunity to develop useful imaging-guided PDT agents for tumor cells.

## Introduction

Photodynamic therapy (PDT) is a promising cancer treatment that has been applied to various cancers, such as oral, lung, bladder, brain, ovarian, and esophageal cancers^1–3^. The PDT process requires three key components: light, oxygen, and a photosensitizing agent^4,5^. In the presence of external light, the photosensitizer (PS) is photoexcited to the optically allowed S_1_ state and then energetically relaxed to the T_1_ state via intersystem crossing (ISC). The triplet excited PS can transfer the excitation energy to ground-state triplet oxygen (type II), resulting in cytotoxic reactive oxygen species (ROS), such as singlet oxygen (^1^O_2_), which directly kills tumor cells^6–9^. PDT can provide good selectivity and is non-invasive at the treatment region as the activity of this medical technique is only carried out when the PS is combined with light of a particular wavelength^10^. Recently, imaging-guided PDT has been assessed to develop specific agents for visualizing individual tumor targets, thereby enhancing therapeutic efficiency and reducing side effects^11–13^. However, to date, PSs that can be simultaneously applied for both imaging and treatment are not available in the clinic. Accordingly, there is an urgent need to develop PSs that can effectively produce both fluorescence and ROS^14,15^.

Among the different PSs, 4, 4-difluoro-4-bora-3a,4a-diaza-s-indacene (BODIPY) is a new family of fluorescent dyes with outstanding photophysical features, such as high molar extinction coefficient, high quantum efficiencies of fluorescence, and easy electronic modification of frontier molecular orbitals by substituents. BODIPYs have thus been applied as promising imaging/detection agents with high light-to-dark toxicity ratios^16–19^. Many dyes with a high ISC obtained from natural or synthetic sources have been employed in PDT reactions^20,21^. The most common design strategy employed to enhance ISC efficiency is the conjugation of heavy halogen atoms (Br or I) to promote spin–orbit coupling (SOC), which improves the ^1^O_2_ generation capability and the population of longer-lived excited triplet states^22,23^. However, the incorporation of heavy halogen atoms causes toxicity and fluorescence quenching^24–26^. Therefore, BODIPY PSs without heavy halogen atoms are preferred as theranostic agents.

Several approaches with heavy-atom-free PSs to improve the ISC, such as the use of dimer BODIPY^27,28^, spin converters^29^, and photoinduced electron transfer (PeT)^30,31^, have recently been reported. The formation of triplet states via photoinduced electron transfer (PeT) is a well-known process that was not employed to develop practical triplet sensitizers until recently^32^. The charge-transfer (CT) states comprised the donor radical cation and the acceptor radical anion are induced by PeT, which recombines the ground state via different pathways^31,33,34^. Owing to the different polarity between locally-excited (LE) and CT states, their relative energy levels are strongly affected by the polarity of medium and accordingly the energy relaxation dynamics much complicated. For example, in non-polar solvents, the energy level of polar CT state is usually higher than that of the LE state, resulting in very low PeT efficiency and intense fluorescence from the LE state. In contrast, for solvents with sufficient polarity, such as in an aquatic environment, the CT state is energetically mush stabilized and thus its energy level can be low lying compared to that of the LE state, causing effective PeT and ISC^35^.

**Figure 1:**
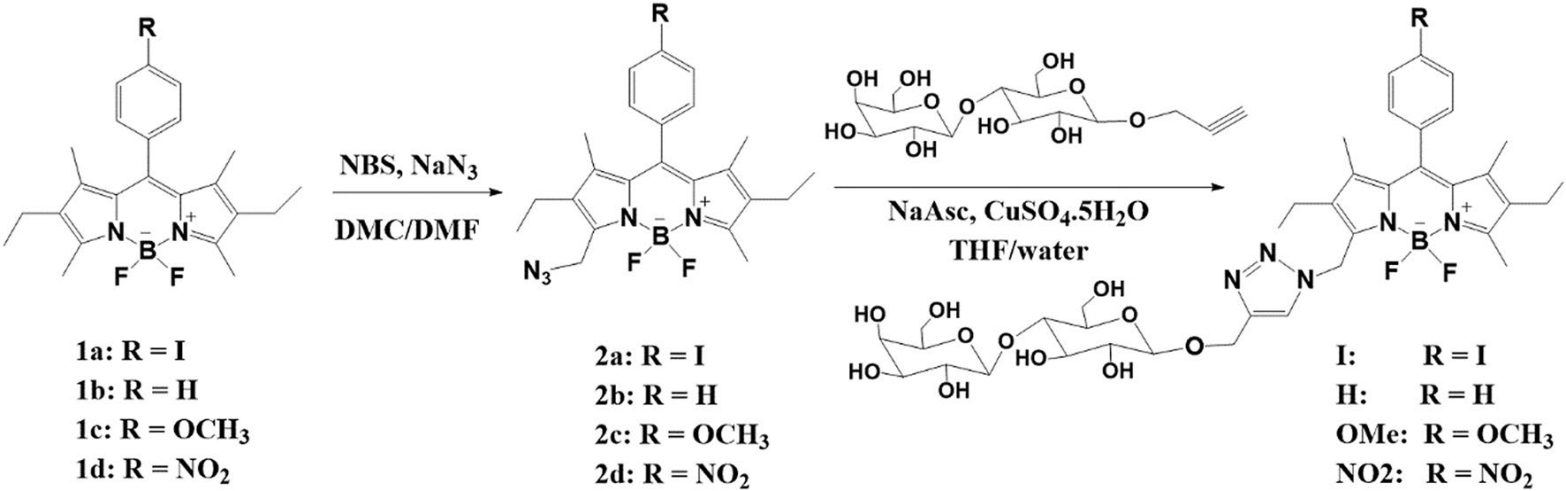
The preparation of lactose-functionalized BODIPY PSs with various *meso*-phenyl substituents.

Recently, we reported a series of water-soluble BODIPY PSs attached to heavy atoms at the 2,6-position of the BODIPY core^24^. These BODIPY PSs showed excellent PDT ability, while exhibiting very low dark toxicity; however, they could not be used as imaging reagents due to their low fluorescence quantum yield (Φ_F_) resulting from the incorporation of heavy atoms. To overcome the above-mentioned issues, we aim to develop bifuntional heavy-atom-free BODIPY PSs with imaging-guided PDT properties. Herein, we present a new family of heavy-atom-free BODIPY nanoparticles (NPs) (Fig. 1) with potential applications in tumor-targeted fluorescence cell imaging and PDT. We synthesized four BODIPY PSs containing different substituent groups (-I, -H, -OCH_3_, and -NO_2_) in the *para*-phenyl moiety attached to the *meso*-position of the BODIPY core, and investigated their photophysical and photosensitizing properties according to the variation in the substituents. Among the BODIPY PSs, we used BODIPY **H** as an internal reference to compare the fluorescence and PDT efficiencies without substituent. In addition, BODIPY segments were connected with lactose-tethering triazole as a specific ligand for asialoglycoprotein (ASGP) in liver cancer cells^36^. The resulting BODIPY PSs formed NPs with a uniform size in water. Further, the cell viability, cellular imaging, and photodynamic anticancer activities of these BODIPY NPs were evaluated using HeLa and Huh-7 cells. Overall, our findings indicate that BODIPY NPs are promising tumor-targeted PDT agents, with fluorescence cell imaging properties in live cancer cell lines.

## Results and discussion

### Synthesis and photophysical characterization of BODIPY NPs

The process used to synthesize the four BODIPY PSs (**I**, **H**, **OMe**, and **NO2**) functionalized with lactose-tethering triazole is outlined in Fig. 1. The detailed procedure is provided in the Supporting Information section.

Compounds **1a–d** were synthesized via condensation reactions using 3-ethyl-2, 4-dimethyl pyrrole with 4-iodobenzoyl chloride and benzaldehyde derivatives, which yielded compounds **1a** and **1b–d**, respectively. Compounds **2a–d** modified with alkyl azide at the 3-methyl position of the BODIPY core were obtained in the same manner^37^. Cu(I)-catalyzed alkyne–azide cycloaddition reactions (CuAAC) were performed with compounds **2a-d** and propargyl lactoside to obtain the final compounds BODIPY **I**, **H**, **OMe**, and **NO2**, respectively. The final BODIPY PSs were fully characterized by ^1^H, ^13^C NMR, and HR-MS, as shown in Fig. S8 -27.

A series of water-dispersible NPs, namely **I-NPs**, **H-NPs**, **OMe-NPs**, and **NO2-NPs**, were obtained from the corresponding BODIPY PSs **I**, **H**, **OMe**, and **NO2** in aqueous solution, respectively, after complete evaporation of tetrahydrofuran (THF).

**Figure 2:**
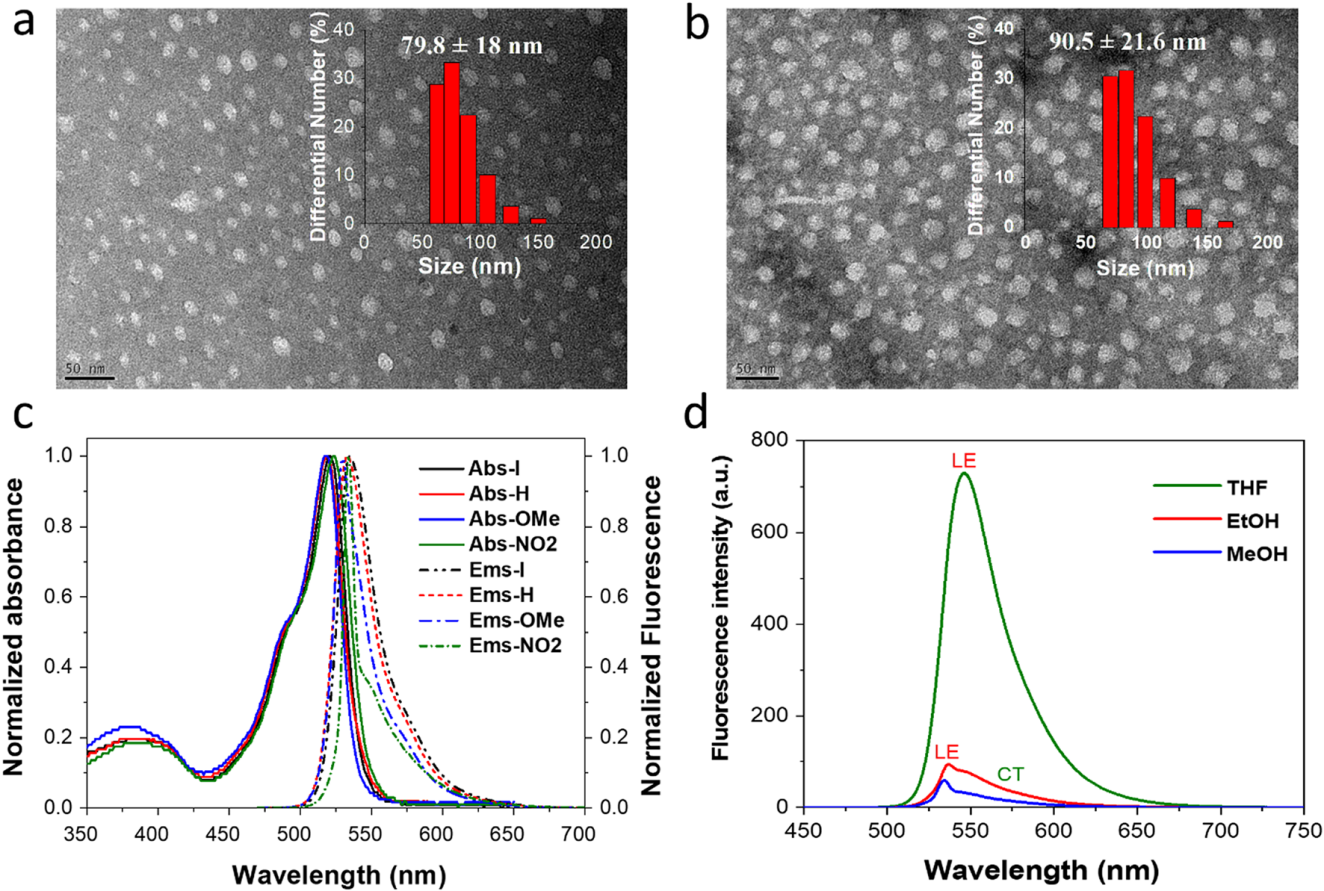
Characterization of BODIPY PSs. TEM images of **I**-**NPs** (**a**) and **OMe**-**NPs** (**b**) (the scale bar: 50 nm). The inset images indicate the sizes of **I-NPs** and **OMe-NPs** based on DLS. The normalized absorption and emission spectra of **I, H, OMe**, and **NO2** in methanolic solution at c = 5 µM (**c**). The emission spectra of BODIPY **NO2** in various solvents at c = 5 µM (**d**). The solutions were excited at 490 nm.

The hydrodynamic diameters of the obtained BODIPY NPs in aqueous solution were determined using dynamic light scattering (DLS) measurements. As shown in Fig. 2a,b and Figure S1, the average diameters of the **H**, **I**, **OMe**, and **NO2**-**NPs** were approximately 71.3, 79.8, 90.5, and 100.7 nm, respectively. The morphology and size of BODIPY NPs were also examined by transmission electron microscopy (TEM), which revealed their spherical morphology and average diameter of approximately 20–30 nm (Figs. 2a and b). Notably, the size of the fully hydrated BODIPY NPs as measured by DLS might be larger than that determined in the dried state from TEM. Many water molecules seem to be entrapped within the BODIPY NPs via interactions with hydrophilic lactose segments.

Figure 2c shows the absorption and fluorescence spectra of the BODIPY PSs in methanol. All of the synthesized BODIPY PSs exhibit typical two absorption bands, with a robust S_0_ → S_1_ (π → π*) transition band around 518–524 nm, an extinction coefficient of 42,400–49,500 M^-1^·cm^-1^ from the boradiazaindacene chromophore^38,39^, and a weak broad band around 350–400 nm corresponding to the S_0_ → S_2_ (π → π*) transition, which can be attributed to the out-of-plane vibrations of the aromatic skeleton^37,40^. The iodine (-I) and methoxy (-OCH_3_) substituents incorporated into the *meso*-phenyl of BODIPY resulted in almost the same absorption and emission spectra as BODIPY **H**, indicating that these substituents have a negligible effect on the photophysical properties of the BODIPY cores^37^. The BODIPY dyes with **H**, **I**, and **OMe** exhibited a high Φ_F_ of approximately 0.45–0.51 (Table 1), which is usually recorded for BODIPY derivatives^37^. Of note, the values of Φ_F_ remained high despite the BODIPY dyes being modified with the lactose-tethering triazole and iodine substituent^24,41^. However, the nitro substituent (-NO_2_) appeared to have a more significant influence on the photophysical properties of the BODIPY dye. Although the absorption spectrum of BODIPY **NO2** is similar to those of the other BODIPY PSs, its fluorescence quantum yield is largely reduced (Table 1) as well as its fluorescence intensity and spectral shape are strongly affected by the solvent polarity (Fig. 2d). In aqueous solution, BODIPY I, H, and OMe-NPs exhibited absorption and emission maxima (λ_abs_/λ_em_) at around 515/538 nm while the fluorescence of NO2-NPs was quenched (Figure S7).

**Table 1:** The photophysical and photosensitizing properties of BODIPY **I**, **H**, **OMe**, and **NO2** in MeOH.

	I	H	OMe	NO_2_
λ_ab_ (nm)^a^	522	518	520	524
λ_em_ (nm)^a^	535	533	530	534
ɛ (10^3^ M^-1^ cm^-1^)^a^	45.2	42.4	44.3	49.5
Φ_F_^b^	0.45	0.51	0.49	0.014
Φ_Δ_^c^	0.1	0.037	0.073	0.009

^a^Absorption and fluorescence data measured using methanol.

^b^Fluorescence quantum yields were determined using rhodamine 6G in methanol as the standard (Φ_f_ = 0.94)^42^.

^c^Singlet oxygen quantum yields were determined by the DPBF bleaching method, using HP in ethanol as a reference (Φ_Δ_ = 0.53)^43^.

As shown in Table S1, the emission intensities and Φ_F_ of **NO2** were largely quenched in protic polar solvents, exhibiting Φ_F_ of 0.035 in ethanol and 0.014 in methanol compared with 0.38 in THF. The intense emission observed in THF corresponds to the fluorescence from the local excited (LE) state of the BODIPY subunit. In contrast, fluorescence is largely quenched in a polar methanol solution, owing to a nearly non-emissive CT state^44^. This phenomenon is expected because the population of non-emissive CT state by PeT mechanism competes with that of emissive LE state^30,33,34^. Details of the photophysical studies are presented in the theoretical calculation section.

### Singlet oxygen generation

The singlet oxygen (^1^O_2_) generation capabilities of **I**, **H**, **OMe,** and **NO2** were assessed in air-saturated ethanol under irradiation at 520 nm. A commercial ^1^O_2_ probe, 1,3-diphenylisobenzofuran (DPBF), was used as an indicator, and hematoporphyrin (HP) in EtOH was used as the reference (*Φ*Δ = 0.53)^45^.

**Figure 3:**
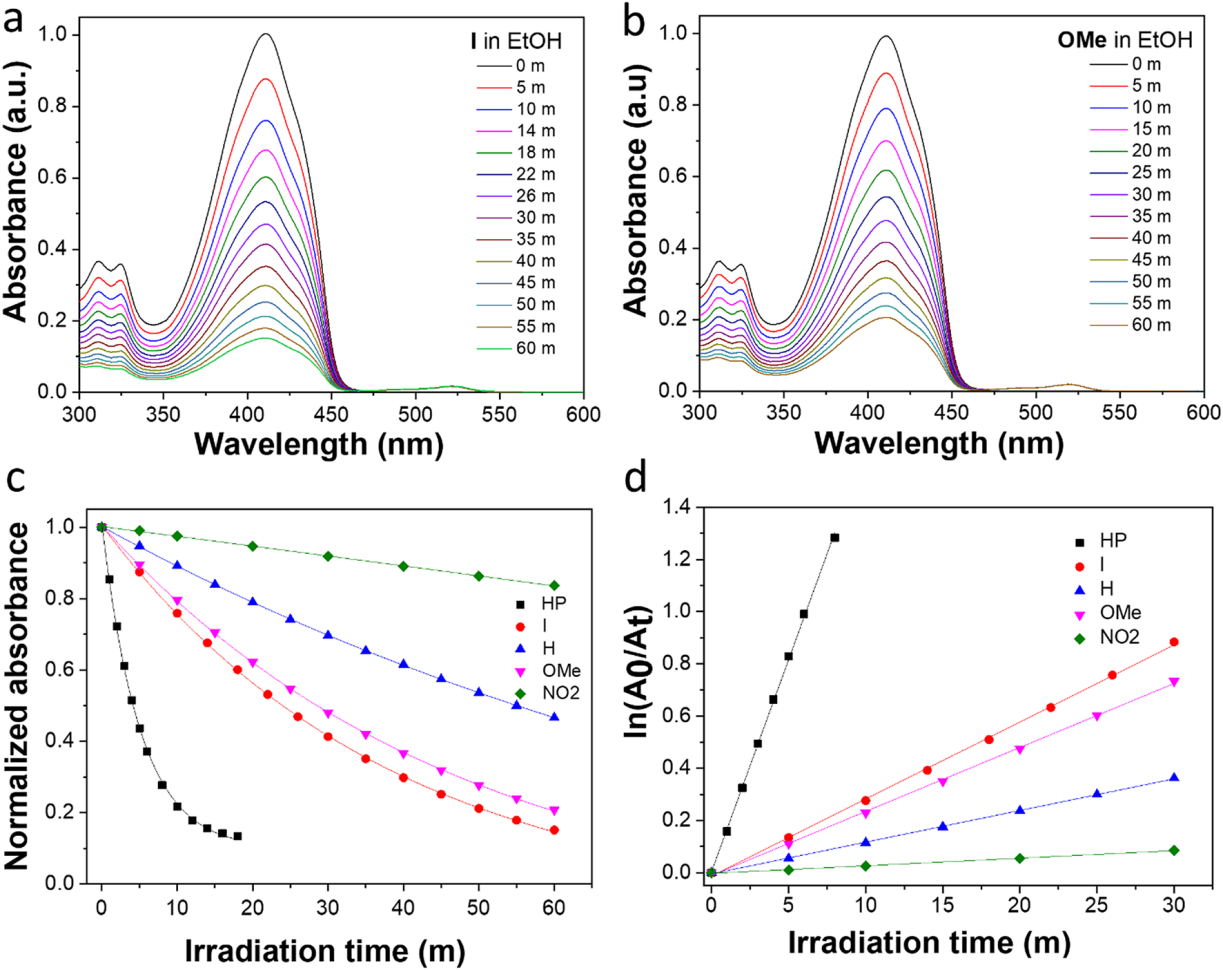
The singlet oxygen **(**^**1**^**O**_**2**_**)** generation capabilities of BODIPY PSs. Absorption spectra of DPBF upon irradiation in the presence of (**a**) **I** and (**b**) **OMe** under 520 nm for different times. (**c**) Plots of the change in absorbance of DPBF at 414 nm at different irradiation times using hematoporphyrin (HP) as the standard in EtOH at room temperature (*Φ*Δ = 0.53). (**d**) ^1^O_2_ assay using the absorbance attenuation of DPBF in the presence of BODIPY **I**, **H**, **OMe**, and **NO2** against HP as the standard in EtOH.

As shown in Figs. 3a–c and S3, the absorbance of DPBF at 414 nm decreased gradually in the presence of the BODIPY dyes under continuous light irradiation. According to the linear relationship of the decay curves (Fig. 3d), the ^1^O_2_ quantum yields (Φ_△_) of **I**, **H**, **OMe,** and **NO2** were assigned as 0.1, 0.037, 0.073, and 0.009, respectively (Table 1). Thus, the most robust ^1^O_2_ generation ability of **I** among the series of PS suggested that the additional heavy iodine atom on the *meso*-phenyl of BODIPY induced spin–orbit perturbations on the molecules and significantly influenced its superior capability to generate singlet oxygen, as shown by the faster reducing rate of DPBF absorbance bands and a higher Φ_△_ than that of the other BODIPY PSs. However, the incorporation of the electron-donating group (-OCH_3_) at the *meso*-phenyl of BODIPY played an essential role in enhancing the ^1^O_2_ generation of **OMe**. In contrast, the introduction of a strong electron-withdrawing group (-NO_2_) did not lead to an efficient Φ_△_ of BODIPY **NO2** in a polar solvent (ethanolic solution). However, as shown in Figure S4a, Φ_△_ could be enhanced to 0.03 in a less polar solvent (THF). Such finding indicates that the triplet state of BODIPY **NO**_**2**_ is strongly affected by the polarity of the media (the details are presented in the theoretical calculation). These results collectively indicate that both iodinated- *meso*-phenyl BODIPY **I** and heavy-free atom BODIPY **OMe** achieved an elevated ^1^O_2_ under LED illumination that facilitated singlet oxygen generation, demonstrating their potential use as efficient photosensitizers for PDT.

### Theoretical characterization of the BODIPY derivatives

**Figure 4:**
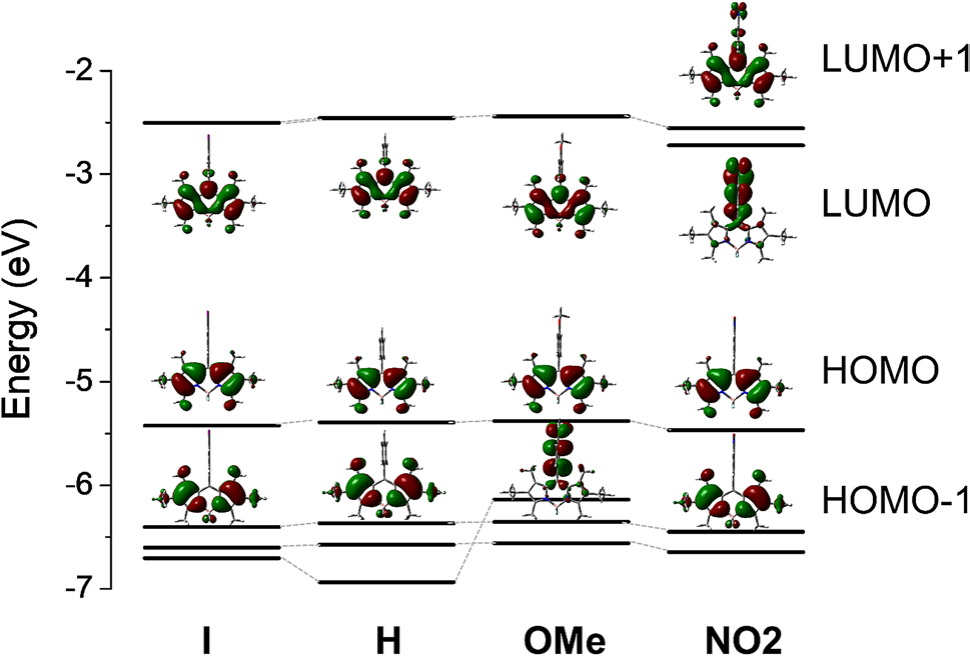
Energy diagram of frontier molecular orbitals of the BODIPY PSs.

To determine the effect of *meso*-phenyl substituents on the electronic structure of BODIPY PSs, density functional theory (DFT) calculations were performed (see Methods for computational details). As depicted in Fig. 4, the HOMO-3 of BODIPY **OMe** is destabilized by electron-donating methoxy substituent and consequently HOMO-1 of BODIPY **OMe** is mainly concentrated on methoxyphenyl (MPh). On the other hand , the LUMO of BODIPY **OMe** is fully concentrated on the BODIPY (BDP) moiety, implying that upon the generation of the singlet excited state by photoexcitation, PeT may occur, leading to the CT state, ^1^[BDP^•-^–MPh^•+^]. This process enhances the triplet state formation efficiency, as demonstrated by the significant increase in ${\Phi }_{\Delta}$ of **OMe**, compared with **H**. The energy level of singlet CT state was approximately 0.2 eV higher in energy than that of the singlet excited state (Table S2). However, Filatov et al. suggested that even if the energy of the CT state is greater than that of the singlet excited state, PeT and the subsequent triplet state formation can occur, with a propensity that the efficiency of the processes reduces with increasing energy gap^30^. Hence, despite the reversal of the state energy, PeT is viewed as a valid mechanism for **OMe**, which facilitates the generation of the triplet state and ^1^O_2_.

The electron-withdrawing nitro group existing in **NO**_**2**_ causes nitrophenyl (NPh) to act as an electron acceptor, while BODIPY becomes an electron donor, as demonstrated by the HOMO and LUMO localized on BODIPY and nitrophenyl, respectively (Figs. 4 and S5). Therefore, in **NO**_**2**_, PeT may produce ^1^[BDP^•+^–NPh^•-^]. Unlike **OMe**, the energy level of the CT state is much lower than that of the LE state of BODIPY moiety with an energy gap of 0.49 eV, and therefore the lowest CT state dominantly contributes to the energy relaxation after photoexcitation (Table S2). The results of the DFT calculations, in conjunction with the solvent dependency of ${\Phi }_{\mathrm{F}}$, clearly confirm the existence of PeT in **NO2**; however, this does not lead to efficient ISC and energy transfer to ground-state ^3^O_2_, as reflected by the very low ${\Phi }_{\Delta}$ of BODIPY **NO2**.

**Figure 5:**
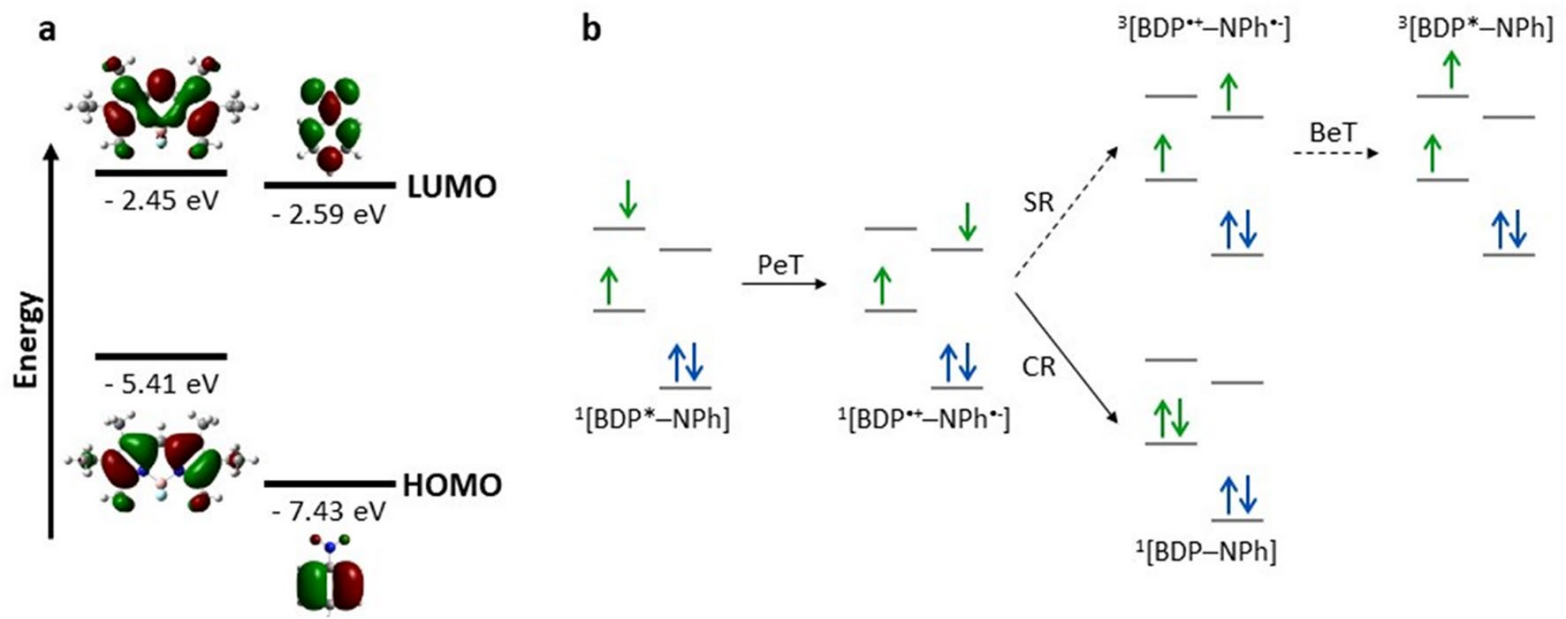
(**a**) Frontier orbital energies of two subunits of NO_2_. (**b**) Schematic of the electron movement required for PeT-mediated triplet state generation in **NO**_**2**_. In (**b**), SR, CR, and BeT represent spin reversion, charge recombination, and back electron transfer, respectively.

For donor–acceptor-type BODIPY dyads, comparing the frontier orbital energies of donor and acceptor units is beneficial for explaining electron movement during the PeT process^44^. This approach applies to **NO2** because, in its optimized geometry, the two subunits are oriented almost perpendicular ( θ_dihedral_ = 87.8°) and can be considered as two independent functional groups. In this context, to elucidate the unexpected decoupling between PeT and the triplet state formation observed for **NO2**, the HOMO/LUMO energies of hexaalkyl-substituted BODIPY and nitrobenzene were calculated and compared, shown in Fig. 5a.

As illustrated in Fig. 5b, ISC from ^1^[BDP^•+^–NPh^•-^] to ^3^[BDP^*^–MPh] requires the back transfer of an electron from LUMO_NPh_ to LUMO_BDP_, following the conversion of its spin; this process is known as the radical pair ISC (RP-ISC). However, DFT calculations revealed that E(LUMO_BDP_) > E(LUMO_NPh_) (Fig. 5a), demonstrating that for **NO2**, back electron transfer (BeT) is an energetically unfavorable process. These findings agree with those of Zhao et al., who explored redox potentials for energetic comparison of frontier orbitals^46^. In addition, electron spin conversion, which must precede the above process, is often forbidden for directly linked donor–acceptor systems, such as **NO2**. Hence, ^1^[BDP^•+^–NPh^•-^] of **NO2** is most likely to decay nonradiatively to the ground state. However, whether this dissipation occurs directly or via the formation of ^3^[BDP–NPh^*^] remains unclear, but it is clear that even if the ^3^[BDP–NPh^*^] mainly formed, it does not contribute to the energy transfer to the ground-state ^3^O_2_.

These results are consistent with those of Qi et al.^47^ The electronic properties of the *meso*-substituent on the BODIPY core, particularly the introduction of a suitable electron-donating group, could be fine-tuned to control the efficiency of singlet oxygen formation. For BODIPY **I**, the perpendicular geometry between BODIPY and iodophenyl moieties prevents strong electronic coupling between BODIPY and iodine, and accordingly BODIPY **I** can maintain high fluorescence quantum yield despite the substitution of heavy-atom iodine. These results suggest that BODIPY **OMe** can be used as a theranostic agent for cancer treatment.

### Cellular uptake and cell-imaging of BODIPY NPs

**Figure 6:**
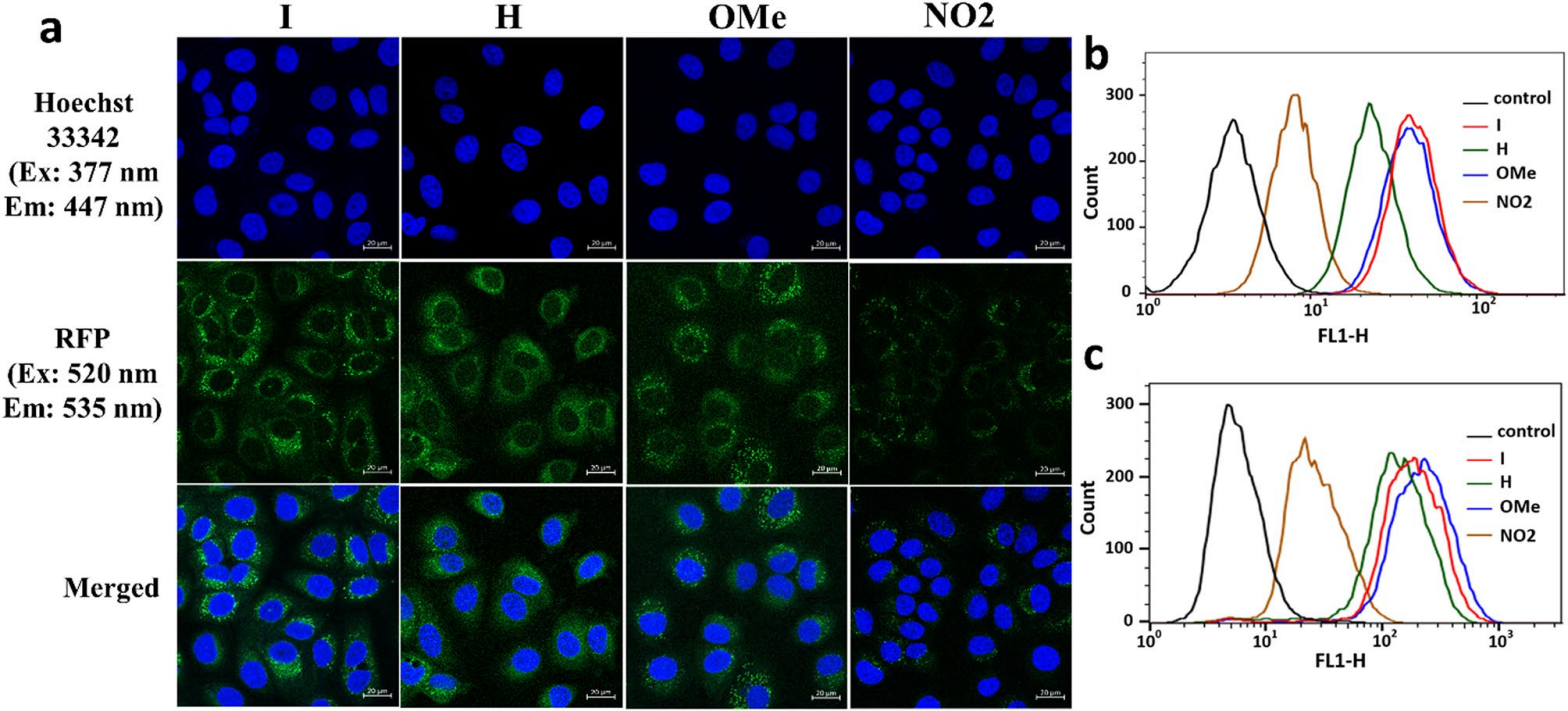
Cell selectivity and imaging of lactose-functionalized BODIPY PSs. Fluorescence images of Huh-7 cells were captured after a 2 h incubation with 2 µM of **I, H, OMe,** and **NO2-NPs**. After incubation, cell nuclei were stained with Hoechst 33,342 dye for 10 min. Images were captured with a 40 × objective lens and fluorescence optics (excitation at 520 nm for **I, H, OMe**, and **NO2-NPs** and 377 nm for Hoechst 33,342; and emission at 535 nm for **I, H, OMe,** and **NO2-NPs** and 447 nm for Hoechst 33,342). Scale bar = 20 μm (**a**). The fluorescence-activated cell sorting (FACS) analysis of HeLa (**b**) and Huh7 cells (**c**) treated with 2 μM of BODIPY **I**, **H**, **OMe**, and **NO2**.The incubation time was 2 h for all BODIPY NPs.

Cellular uptake behaviors of the BODIPY NPs against Huh-7 (human liver carcinoma) and HeLa (human cervix adenocarcinoma) cells were measured and compared by flow cytometry analysis. First, the cells were incubated with 2.0 μM of the BODIPY PSs for 2 h at 37 °C; then, the treated cells were collected and subjected to fluorescence-activated cell sorting (FACS). As depicted in Figs. 6b,c, these BODIPY NPs exhibited higher selectivity for Huh-7 cells than HeLa cells. The mean fluorescence of liver cancer cells treated with **I**, **H**, **OMe**, and **NO2**-**NPs** was approximately 4.6-, 6.2-, 6.1-, and fourfold higher than that of the treated HeLa cells (Fig. 6b,c and Table S3). Such finding is due to the high density of ASGP receptors on the surface of liver cancer cells, which can bind BODIPY NPs containing galactose residues and be successfully internalized into Huh-7 cells via receptor recognition^48,49^, which is consistent with our previous report^24^. This result demonstrates that BODIPY PSs with versatile functional groups can have an improved targeting ability compared to imaging-guided PDT agents which cannot be chemically modified such as 5-aminolevulinic acid (ALA)^50^.

Lactose modification can provide BODIPY NPs with potential applications in specific liver cancer imaging ability. To evaluate the potential application of the fluorescent dye for cell imaging, liver cancer Huh-7 cells were treated with these dyes for 2 h and imaged by confocal microscopy. Hoechst 33,342, a fluorescent stain commonly used to visualize the nucleus, was used to confirm fluorescent dye localization within the cells. As shown in the cellular images (Fig. 6a), the targeting BODIPY NPs could be effectively transported into Huh-7 cells, which was mediated by galactose receptors, after cultivation for 2 h and firmly gathered in the cytoplasm and perinuclear region. Hoechst 33,342 resulted in blue fluorescence in the nucleus of Huh-7 cells, and the merged image revealed that these BODIPY NPs could specifically bind to Huh-7 cells and localize in their cytoplasm. Therefore, these BODIPY NPs can effectively distinguish the cytoplasm of Huh-7 cells from the nucleus.

Huh-7 cells cultured with **H**, **I**, and **OMe-NPs** showed stronger fluorescence, whereas those cultured with **NO2**-**NPs** displayed weaker fluorescence; this is caused by the high fluorescence of H, I, and OMe-NPs along with cellular uptake of **NO2-NPs** being the lowest, consistent with the FACS results. Additionally, as mentioned above, **NO2-NPs** exhibited PeT in polar media, resulting in the CT state, which inhibits fluorescence. As a result, **NO2-NPs** appeared darker in the cell-imaging than the other compounds.

### Light-induced cytotoxicity of BODIPY NPs against cancer cells

**Figure 7:**
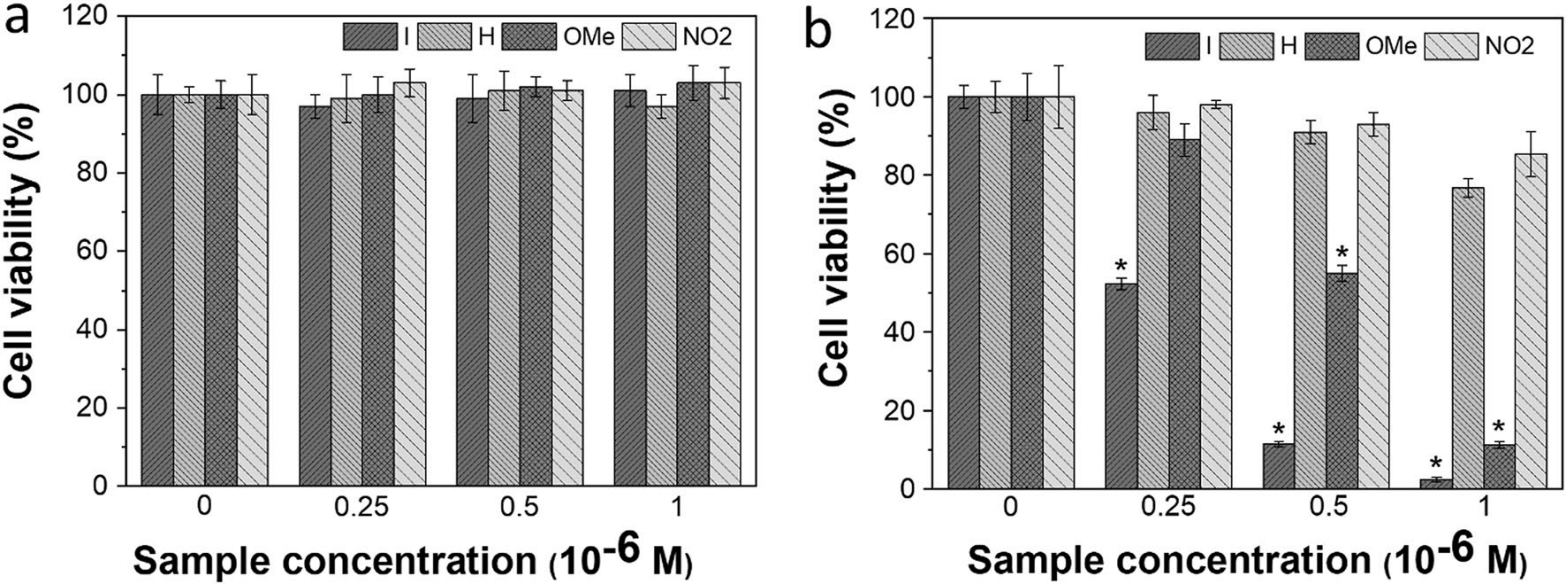
Photodynamic anticancer activities of BODIPY PSs in Huh-7 cells. Cytotoxicity (**a**) and phototoxicity (**b**) of BODIPY **I, H, OMe,** and **NO2-NPs** in Huh-7 cells (hepatocellular carcinoma) under light irradiation (λ_irr_ 530 nm, 8.6 J·cm^-2^). The cell viabilities were detected using a CCK-8 kit after incubation with **I, H, OMe,** and **NO2-NPs** for 24 h under dark conditions. Quantitative data are expressed as mean ± standard deviation (n = 4). Statistical significance based on the Student’s *t*-tests was considered as *p < 0.05.

The biocompatibility of **I**, **H**, **NO**_**2**_, and **OMe-NPs** with Huh-7 and HeLa cells was determined using MTS assays. As shown in Figs. 7a and S6a, there was no cytotoxicity in any of the tested cells, and more than 97% of both Huh-7 and HeLa cells survived after 24 h of incubation. Thus, all water-soluble BODIPY PSs below a dose of 1 µM had good biocompatibility and did not induce severe cytotoxicity in fibroblasts and cancer cells, implying that BODIPY **I**, **H, NO**_**2**_, and **OMe-NPs** could be used in the light cytotoxicity test.

For the efficient production of ROS, HeLa and Huh-7 cells were exposed to 530 nm laser irradiation at an extremely low energy of 8.6 J·cm^-2^, and the in vitro phototoxicity of PDT was assessed. First, there was no significant variance when HeLa and Huh-7 cells were cultured with RPMI containing 1 μM of **H** and **NO**_**2**_**-NPs**, not only under dark conditions but also under irradiation; this is because of their low singlet oxygen quantum yield, as mentioned above. When exposed to LED light, **I** and **OMe**-**NPs** resulted in high phototoxicity to tumor cells and negligible cell toxicity in the dark, as shown in Figs. 7b and S6b. Cell viability evidently decreased as the concentration of these BODIPY NPs increased. Furthermore, when HeLa cells were exposed to light in the presence of 1 μM of **OMe** and **I-NPs**, cell viability decreased by approximately 78% and 83%, respectively, while Huh-7 cells died by up to 89% and 97.7%, respectively, indicating their efficient PDT targeting ability. Besides, the half-lethal dose (IC50) of **OMe-NPs** for HeLa and Huh-7 cell lines was 0.62 and 0.52 μM, respectively. Notably, when exposed to light, the lactose-tethered BODIPY **OMe** and **I-NPs** killed more Huh-7 cells than HeLa cells, with IC50 values of 0.51 and 0.26 μM, respectively. These results demonstrate that BODIPY **I** and **OMe-NPs** provide better treatment efficacy with relatively low irradiation intensity than **H** and **NO2-NPs**.

### Conclusion

We designed and synthesized a series of lactose-functionalized BODIPY PSs with different substituent groups at the *meso*-position of the BODIPY core as cancer-targeted theragnostic agents for imaging and PDT. These BODIPY PSs could aggregate into nanoparticles (**I-, H-, OMe-,** and **NO**_**2**_**-NPs**) in an aqueous solution and displayed a uniform size and shape, as determined by TEM and DLS. The fluorescence quantum yields of BODIPY **I**, **H**, and **OMe** were remarkably high, whereas that of BODIPY **NO2** was significantly lower due to the PeT process in polar media. Among the four BODIPY photosensitizers, BODIPY **I** demonstrated high efficiency of singlet oxygen generation caused by the heavy atom effect due to the presence of an iodine atom, while BODIPY **OMe** containing an electron-donating methoxy group at the *meso*-phenyl moiety also enhanced the ISC efficiency. In contrast, the strong electron withdrawing by nitro group (NO_2_) caused a marked reduction in both the fluorescence quantum yield and singlet oxygen formation efficiency of BODIPY **NO2** due to effective PeT in the polar media. The cellular experiments demonstrated that the water-soluble BODIPY NPs series showed good biocompatibility and cancer-specific fluorescence imaging ability. Notably, BODIPY **OMe** and **I-NPs** presented excellent phototoxicity against cancer cells, especially, liver cancer Huh-7 cells. The biocompatible BODIPY NPs have proven to be promising for developing highly efficient theragnostic agents for imaging-guided PDT for cancer treatment.

## Methods

### Materials instrumentations

Almost all reagents and chemicals were obtained from Sigma Aldrich (St. Louis, MO, USA). Some solvents such as dichloromethane (CH_2_Cl_2_), methanol (MeOH), or MgSO_4,_ sodium azide (NaN_3_), sodium ascorbate (NaAsc), Copper (II) sulfate pentahydrate (CuSO_4_.5H_2_O) were purchased from Daejung chemical (Gyeonggi-do, South Korea), and used without further purification. Lactose-propargyl was synthesized in our previous literature^24^.

All compounds were characterized by ^1^H and ^13^C-NMR spectroscopy on a Bruker AM 250 spectrometer (Billerica, MA, USA). The impurity of the products was checked by thin-layer chromatography (TLC, silica gel 60 mesh). UV spectra were measured on a Shimadzu UV-1650PC spectrometer, and Fluorescence spectra were carried on a Hitachi F-7000 spectrometer. The size and morphology of BODIPY NPs were analyzed by using dynamic light scattering (DLS) on Malvern Zetasizer Nano ZS90 and transmission electron microscopy (TEM). We used machine JEOL- JEM 2100F at an accelerating voltage of 200 kV. The sample for TEM was prepared according to our reported literature^51^.

### Synthesize of water-soluble BODIPY I, H, OMe, and NO2:

According to our reported literature^24^, the series of water-soluble BODIPY **I**, **H**, **OMe**, and **NO2** were prepared using the same pathway. A representative routine is presented for the compound **I**. Briefly, BODIPY **2a** (80 mg, 0.157 mmol), lactose propargyl (66 mg, 0.173 mmol), NaAsc (156 mg, 0.785 mol), and CuSO_4_.5H_2_O (79 mg, 0.316 mmol) were dissolved in the mixture of THF/water (15/5 mL, v/v). The resulting mixture was stirred for one day at room temperature, extracted with THF and water three times, and dried over MgSO_4_. After removing the solvent by a rotary evaporator, the crude product was purified by recrystallization using MeOH/diethyl ether to afford an orange solid (yield 76 mg, 52% yield). ^1^H NMR (300 MHz, CD_3_OD, δ, ppm): δ 8.02 (s, 1H), 7.97- 7.95 (d, 2H), 7.17- 7.15 (d, 2H), 5.83 (s, 2H), 4.36- 4.33 (d, 2H), 3.87- 3.85 (d, 2H), 3.81- 3.80 (d, 2H), 3.76- 3.74 (d, 1H), 3.71- 3.69 (d, 1H), 3.57- 3.53 (d, 2H), 3.49- 3.48 (d, 2H), 3.31- 3.29 (d, 2H), 2.57 (s, 3H), 2.42–2.39 (q, 4H), 1.44 (s, 3H), 1.36 (s, 3H), 1.28 (s, 3H), 1.05- 1.00 (t, 3H). ^13^C NMR (75 MHz, CD_3_OD, δ, ppm): δ 162.25, 143.55, 143.24, 142.81, 140.13, 138.60, 137.3, 135.75, 134.58, 134.16, 131.48, 131.32, 105.09, 103.29, 96.18, 80.57, 77.05, 76.45, 76.29, 74.75, 74.58, 72.52, 70.38, 63.00, 62.42, 61.82, 46.12, 17.57, 17.32, 14.87, 14.76, 13.2, 12.45, 11.92. HRMS (ESI): calculated for (C_38_H_49_BF_2_IN_5_O_11_): m/z: [M]: 928.2613; found: 928.2619.

**Compound H:** BODIPY **H** was synthesized according to the general procedure to afford the orange solid (67 mg, 57% yield). ^1^H NMR (300 MHz, CD_3_OD, δ, ppm): δ 8.01 (s, 1H), 7.59- 7.57 (t, 3H), 7.37- 7.36 (d, 2H), 5.84 (s, 2H), 4.37- 4.33 (d, 2H), 3.87- 3.85 (d, 2H), 3.81- 3.80 (d, 2H), 3.76- 3.73 (d, 1H), 3.69- 3.66 (d, 1H), 3.59- 3.57 (d, 2H), 3.54- 3.52 (d, 2H), 3.49- 3.32 (d, 2H), 2.53 (s, 3H), 2.43–2.38 (q, 4H), 1.38 (s, 3H), 1.31 (s, 3H), 1.28 (s, 3H), 1.04- 0.99 (t, 3H). ^13^C NMR (75 MHz, CD_3_OD, δ, ppm): δ 161.82, 144.19, 143.61, 143.46, 138.71, 136.45, 134.29, 131.7, 130.75, 130.61, 129.43, 105.16, 103.36, 80.45, 77.26, 77.15, 76.41, 74.6, 72.4, 70.26, 62.96, 62.53, 61.77, 61.66, 48.07, 17.68, 14.85, 14.5, 13.16, 12.24, 11.66**.** HRMS (ESI): calculated for (C_38_H_55_BF_2_IN_5_O_12_Na): m/z: [M + Na]^+^: 834.3713; found: 834.3714.

**Compound OMe:** BODIPY **OMe** was synthesized according to the general procedure to afford the orange solid (55 mg, 47% yield). ^1^H NMR (300 MHz, CD_3_OD, δ, ppm): δ 8.0 (s, 1H), 7.23- 7.2 (d, 2H), 7.13- 7.1 (d, 2H), 5.87 (s, 2H), 4.37- 4.34 (d, 2H), 3.89 (s, 3H), 3.77- 3.71 (q, 3H), 3.6- 3.5 (m, 6H), 3 42- 3.39 (d, 1H), 2.56 (s, 3H), 2.4–2.38 (q, 4H), 1.43 (s, 3H), 1.36 (s, 3H), 1.28 (s, 3H), 1.01- 0.95 (t, 3H). ^13^C NMR (75 MHz, CD_3_OD, δ, ppm): δ 162.15, 144.48, 143.03, 138.59, 136.87, 134.71, 134.18, 132.17, 130.55, 128.29, 116.06, 105.04, 103.33, 80.37, 77.08, 76.53, 76.22, 74.82, 74.64, 72.64, 70.21, 63.04, 62.43, 61.75, 55.8, 46.14, 17.73, 17.44, 14.87, 14.26, 13.28, 12.53, 11.56**.** HRMS (ESI): calculated for (C_39_H_52_BF_2_N_5_O_12_Na): m/z: [M + Na]^+^: 854.3571; found: 854.3572.

**Compound NO2:** BODIPY **NO2** was synthesized according to the general procedure to afford the orange solid (80 mg, 61% yield). ^1^H NMR (300 MHz, CD_3_OD, δ, ppm): δ 8.47- 8.44 (d, 2H), 8.01 (s, 1H), 7.69- 7.66 (d, 2H), 5.83 (s, 2H), 4.37- 4.34 (d, 2H), 3.86- 3.32 (d, 2H), 3.79- 3.75 (d, 2H), 3.6- 3.5 (m, 6H), 3.31- 3.29 (d, 2H), 2.59 (s, 3H), 2.41- 2.39 (q, 4H), 1.39 (s, 3H), 1.31 (s, 3H), 1.29 (s, 3H), 1.04- 0.98 (t, 3H). ^13^C NMR (75 MHz, CD_3_OD, δ, ppm): δ 163.11, 149.96, 143.16, 141.37, 138.46, 137.78, 134.63, 133.69, 131.64, 130.96, 125.76, 105.14, 103.6, 80.37, 77.35, 76.4, 76.24, 74.84, 74.35, 72.54, 70.36, 63.11, 62.44, 61.76, 46.06, 19.39, 17.86, 14.86, 14.46, 13.09, 12.63, 11.71**.** HRMS (ESI): calculated for (C_38_H_49_BF_2_N_6_O_13_Na): m/z: [M + Na]^+^ : 869.3316; found: 869.3318.

### Measurement of photophysical properties

The Φ_f_ were measured by a comparative method using the standard reference with the already-known value of Φ_f_ as the following Eq. (1):

1 $$ {\Phi }_{S} = {\Phi }_{R} \left( {\frac{{A_{R} }}{{A_{S} }}} \right)\left( {\frac{{n_{S} }}{{n_{R} }}} \right)^{2} \left( {\frac{{D_{S} }}{{D_{R} }}} \right) $$

The subscript of S and R represent sample and reference, respectively. N is the refractive index of solvent. A and D are the absorbance and the integrated fluorescence area, respectively. Solutions should be optically dilute to avoid inner filter effects. The Rhodamine 6G was used as the reference sample, which possessed a known quantum yield of 0.94^42^.

### Detection of singlet oxygen quantum yields

The quantum yields of singlet oxygen (Φ_Δ_) of BODIPY **I, H, OMe, and NO2** were studied using diphenylisobenzofuran (DBPF) as a chemical quencher^43^. Briefly, a mixture of the BODIPY dye (absorption ~ 0.02 at 520 nm in EtOH) and the DPBF (absorption ~ 1.0 at 414 nm in EtOH) was irradiated with a laser light (λ_irr_ = 520 nm). The photooxidation of DPBF was monitored between 0 ~ 60 min depending on the efficiency of the PSs. The singlet oxygen quantum yield was calculated using hematoporphyrin (HP) as the reference with a yield of 0.53 in ethanol according to the following Eq. (2):

2 $$ {\Phi }_{\Delta }^{S} = {\Phi }_{\Delta }^{R} \times \frac{{k_{S} }}{{k_{R} }} \times \frac{{{\text{F}}_{{\text{R}}} }}{{{\text{F}}_{{\text{S}}} }} $$

where ${\Phi }_{\Delta }^{R}$ is the singlet oxygen quantum yield of the reference, *k* is the slope of the photodegradation rate of DPBF, S means the sample, R represents the reference. F denotes the absorption correction factor, given by F = 1 – 10^-OD^ (OD at the irradiation wavelength).

### Quantum chemical calculations

Molecular structure optimizations for BODIPY derivatives were carried out using density functional theory (DFT), and their electronic states were calculated using time-dependent DFT (TD-DFT). For **H**, **OMe**, and **NO2**, the b3lyp functional of the Gaussian 16 program package and 6-31G(d) basis sets were chosen. For **I**, due to the heavy iodine atom, lanl2dz basis sets were chosen instead. All calculations were carried out in water solvent environment. The lactose-tethered triazole moiety attached to the position of the BODIPY core was confirmed to have a negligible influence on calculation results (Figure S2) and therefore was replaced by a hydrogen atom for simplicity.

### Preparation of BODIPY nanoparticles (NPs)

The stock solution of each BODIPY dyes in THF (0.5 mg.ml^-1^) was prepared, then 50 µL of stock solution was slowly added to 5 mL of water. The mixture was stirred overnight to evaporate all of THF naturally to yield the designed nanoparticles for further experiment.

### Cells and cell culture

HeLa (human cervix adenocarcinoma), Huh7 (human liver carcinoma) cells were obtained from the Korean Cell Line Bank. The cells were maintained in RPMI 1640 medium (Gibco, Carlsbad, CA, USA) supplemented with 10% heat-inactivated fetal bovine serum (FBS) and antibiotics (100 U/mL penicillin and 100 mg/mL streptomycin) at 37 ºC in a humidified 5% CO_2_ incubator.

### Cell proliferation assay

Cell proliferation was studied using CellTiter 96 ® Aqueous One Solution Cell Proliferation Assay (Promega, Madison, WI, USA) according to the manufacturer’s instructions. Briefly, HeLa and Huh7 (3 × 10^3^ cells/well) were seeded in 96-well plates. After the cells were maintained for 24 h, cells were treated with BODIPY **I, H, OMe, and NO2** at different concentrations (0, 0.25, 0.5, and 1 µM) for 24 h. Following 24 h of incubation, 20 µL MTS [3-(4, 5-dimethylthiazol-2-yl)-5-(3-carboxymethoxyphenyl)-2-(4-sulfophenyl)-2H-tetrazolium] reagent were added to each well and incubated for 4 h at 37 °C. The absorbance was determined at 490 nm using an ELISA plate reader (Thermo Fisher Scientific, Inc., Waltham, MA, USA).

### Assessment of cellular uptake and cellular imaging

To confirm the cellular uptake and cellular imaging by using the BODIPY **I, H, OMe, and NO2,** Huh-7 cells were incubated with 2 µM of BODIPY **I, H, OMe, and NO2** for 2 h and washed three times with DPBS. After then, the cells were subsequently counterstained with Hoechst 33,342 for 10 min. After washing three times with DPBS, the morphologies of Huh-7 cells were taken by an automated live-cell imager (Lionheart FX, BioTek Instruments, Inc., VT, USA) with 40 × objective lens and fluorescence optics (excitation at 520 nm for BODIPY **I, H, OMe, and NO2** and at 377 nm for Hoechst 33,342, and emission at 535 nm for BODIPY **I, H, OMe, and NO2** and at 447 nm for Hoechst 33,342). Cellular images were analyzed using Gen5™ imager software (Ver.3.04, BioTek Instruments, Inc., VT, USA).

### Photodynamic anticancer activity assessment

The HeLa and Huh7cells were seeded at 3 × 10^3^ cells/well in a 96-well plate and incubated at 37 °C in 5% CO_2_. After 24 h, the cells were incubated again with various concentrations of BODIPY **I, H, OMe,** and **NO2** (0, 0.25, 0.5, and 1 µM) at 37 °C in 5% CO_2_ for 2 h under dark conditions. After 2 h incubation for uptaking the BODIPY **I, H, OMe,** and **NO2** into the cells, the media in all plates were changed with RPMI 1640 media without phenol red. Irradiation of cells was performed with a green light-emitting diode (LED) using about 9 mW (530 nm, for 20 min, 80%). After irradiation, the cells were incubated for an additional 24 h, and the cell proliferation was measured using CellTiter 96 ® Aqueous One Solution Cell Proliferation Assay as the same method for the cytotoxicity described above.

### Cellular uptake using flow cytometry

The cells (HeLa and Huh7) were seeded at 1 × 10^5^ cells/well in 6-well plate. After 24 h incubation at 37℃ in 5% CO_2_, the BODIPY **I, H, OMe,** and **NO2** (2 μM) were treated with cells for 2 h. Then, the cell were washed with phosphate buffered saline and analyzed using flow cytometry FC500 (Beckman coulter, CA, USA).

## Statistical analysis

All results are expressed as the means ± standard deviations and were compared by one-way analysis of variance (ANOVA followed by Tukey’s analysis with Prism GraphPad 6 software (San Diego, CA, USA). A significance level was set at *p < 0.05.

## Supplementary Information

Supplementary Information.
