# Supplementary material for: BODIPY nanoparticles functionalized with lactose for cancer-targeted and fluorescence imaging-guided photodynamic therapy
Source: Sci Rep. 2022 Feb 15;12:2541. doi: 10.1038/s41598-022-06000-5 (PMC8847361; doi:10.1038/s41598-022-06000-5)
Supplement: Supplementary file 1 — Supplementary Information. [file 41598_2022_6000_MOESM1_ESM.pdf]

Supporting Information

BODIPY Nanoparticles Functionalized with Lactose for Cancer-Targeted and Fluorescence Imaging-guided Photodynamic Therapy

Duy Khuong Mai[1,2,†], Chanwoo Kim[3,†], Joomin Lee[4,†], Temmy Pegarro Vales[5], Isabel Wen Badon[1], Koushitak De[6], Sung Cho[2,*], Jaesung Yang[3,*], Ho-Joong Kim[1,*]

[1] Department of Chemistry, Chosun University, Gwangju 61452, Korea

[2] Department of Chemistry, Chonnam National University, Gwangju 61186, Korea

[3] Department of Chemistry, Yonsei University, Wonju, Gangwon 26493, Korea

[4] College of Food and Nutrition, Chosun University, Gwangju 61452, Korea

[5] Department of Natural Sciences, Caraga State University, Butuan City 8600, Philippines

[6] Department of Cellular & Molecular Medicine, College of Medicine, Chosun University, Gwangju 61452, Korea

***** Correspondence: scho@chonnam.ac.kr, jaesung.yang@yonsei.ac.kr, and hjkim@chosun.ac.kr

[†] These authors equally contributed to this work.

Received: 21 August 2021; Accepted: 19 January 2022

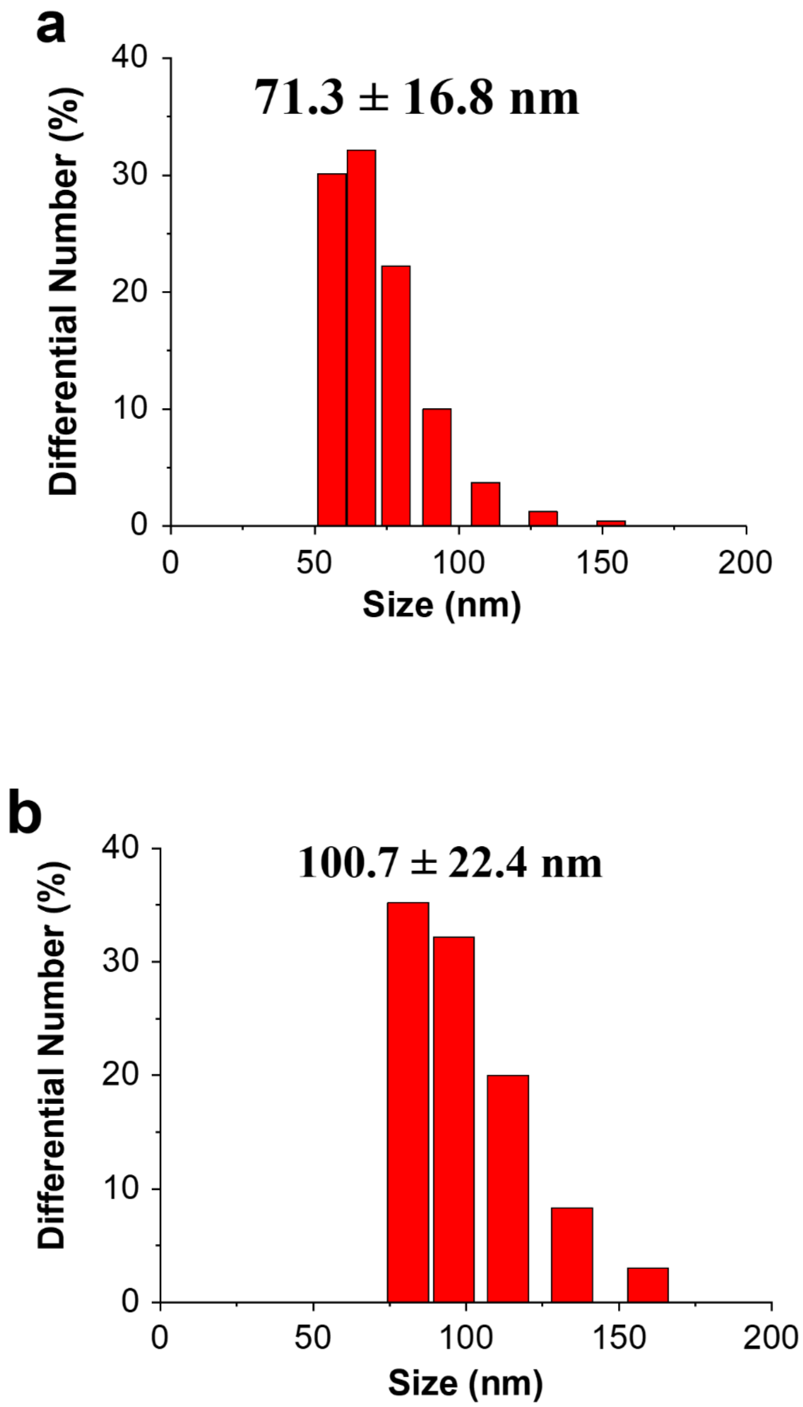

Figure S1. Nanoparticle's size of (a) BODIPY **H** and (b) **NO2** detected by DLS

Table S1. The photophysical properties of compounds **NO2** in various solvents

Sample	Solvent	λ_{ab} (nm)	λ_{em} (nm)	Φ_F
	**THF**	528	546	0.38
**BODIPY NO2**	**EtOH**	525	537	0.035
	**MeOH**	524	534	0.014

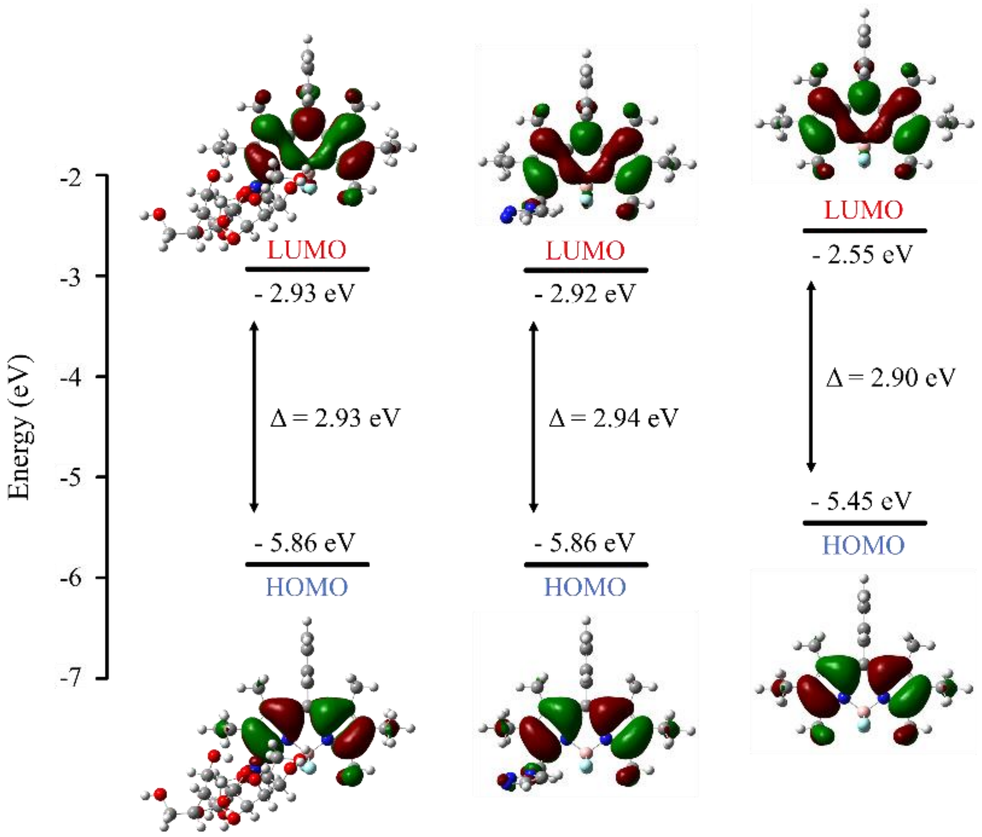

Figure S2. Frontier molecular orbitals of the BODIPY **H**. The complete structure is shown on the left, whereas, in the middle and right, lactose and lactose-tethering triazole substituents are replaced by a hydrogen atom, respectively.

Table S2. Transition energy (E), wavelength (λ), and oscillator strength (f) for the two lowest singlet excited state of BODIPY PSs and the contribution of frontier orbitals to each transition.

	State	E (eV)	λ (nm)	f	Major contribution to transition
**I**	S_1 (LE)	2.78	447	0.5218	H → L (90%), H – 1 → L (10%)
	S_2 (LE)	3.30	375	0.2566	H – 1 → L (89%), H → L (11%)
**H**	S_1 (LE)	2.80	442	0.5715	H → L (92%), H – 1 → L (8%)
	S_2 (LE)	3.30	375	0.1742	H – 1 → L (92%), H → L (8%)
**OMe**	S_1 (LE)	2.80	443	0.569	H → L (93%), H – 2 → L (7%)
	S_2 (CT)	3.00	413	0	H – 1 → L (100%)
**NO2**	S_1 (CT)	2.29	542	0	H → L (100%)
	S_2 (LE)	2.78	446	0.5668	H → L + 1 (93%), H – 1 → L + 1 (7%)

Table S3. Corrected median fluorescence intensities (MFI) of BODIPY **I**, **H**, **OMe**, and **NO2** against treated cancer cell lines

	**I** (log)[a]	**H** (log)[a]	**OMe** (log)[a]	**NO2** (log)[a]
**HeLa**	4336.3 ± 131	2461 ± 34.3	4124 ± 111.3	829 ± 13
**Huh-7**	19864.7 ± 1188.7	15174.7 ± 692.2	25280.7 ± 2181	3341 ± 512

[a] Data are mean ± SD (n = 3)

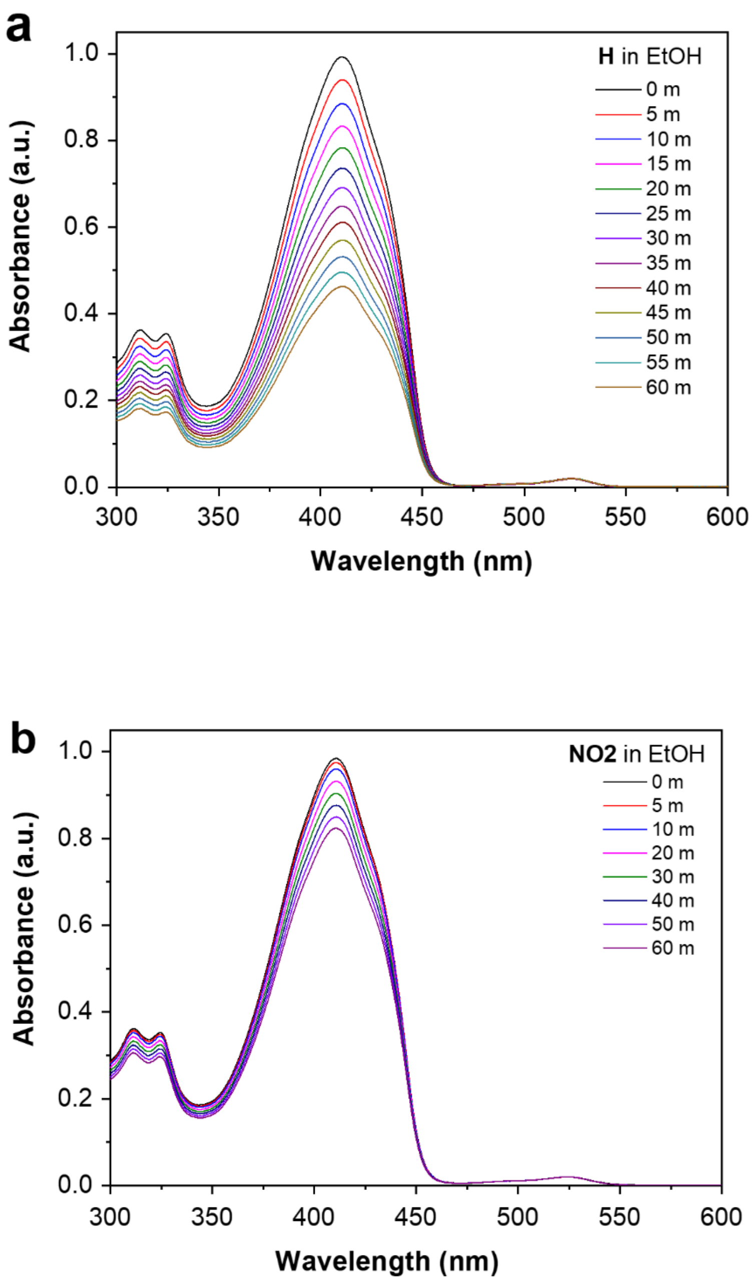

Figure S3. Absorption spectra of DPBF upon irradiation in the presence of (a) BODIPY **H** and (b) **NO2** in EtOH under 520 nm for different times.

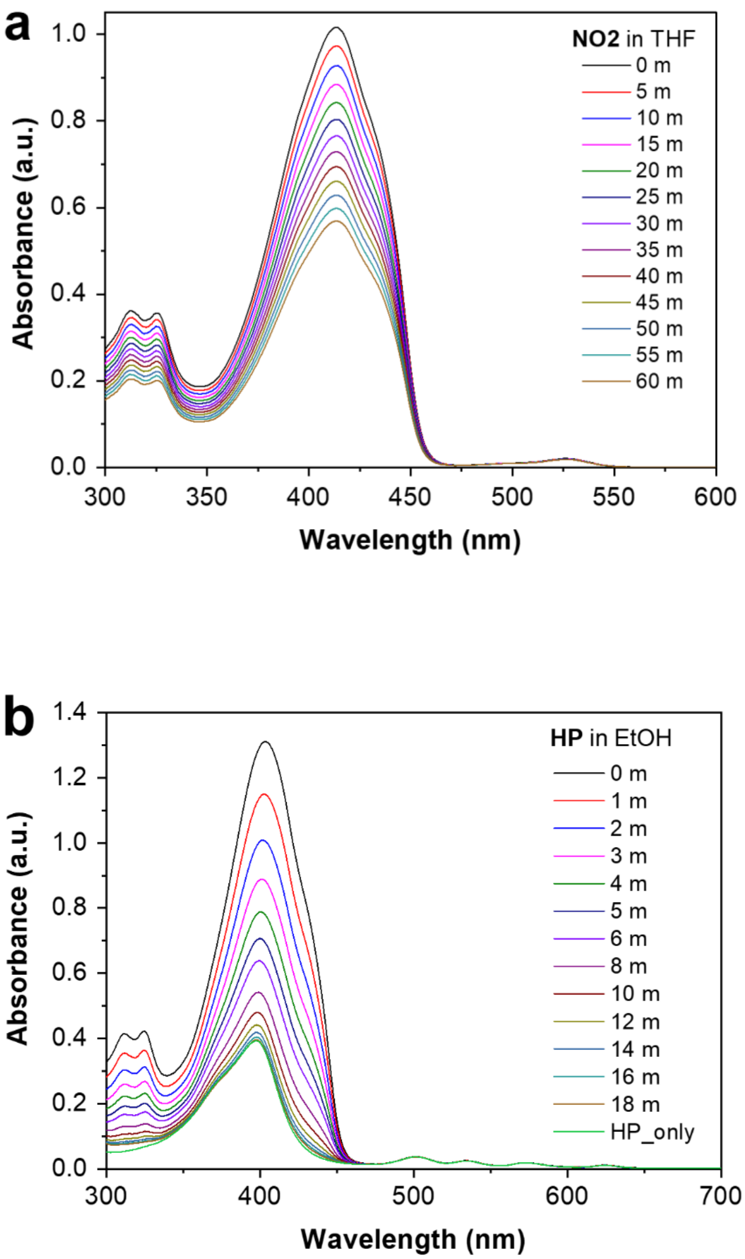

Figure S4. Absorption spectra of DPBF upon irradiation in the presence of (a) **NO2** in THF and (b) **HP** in EtOH under 520 nm for different times.

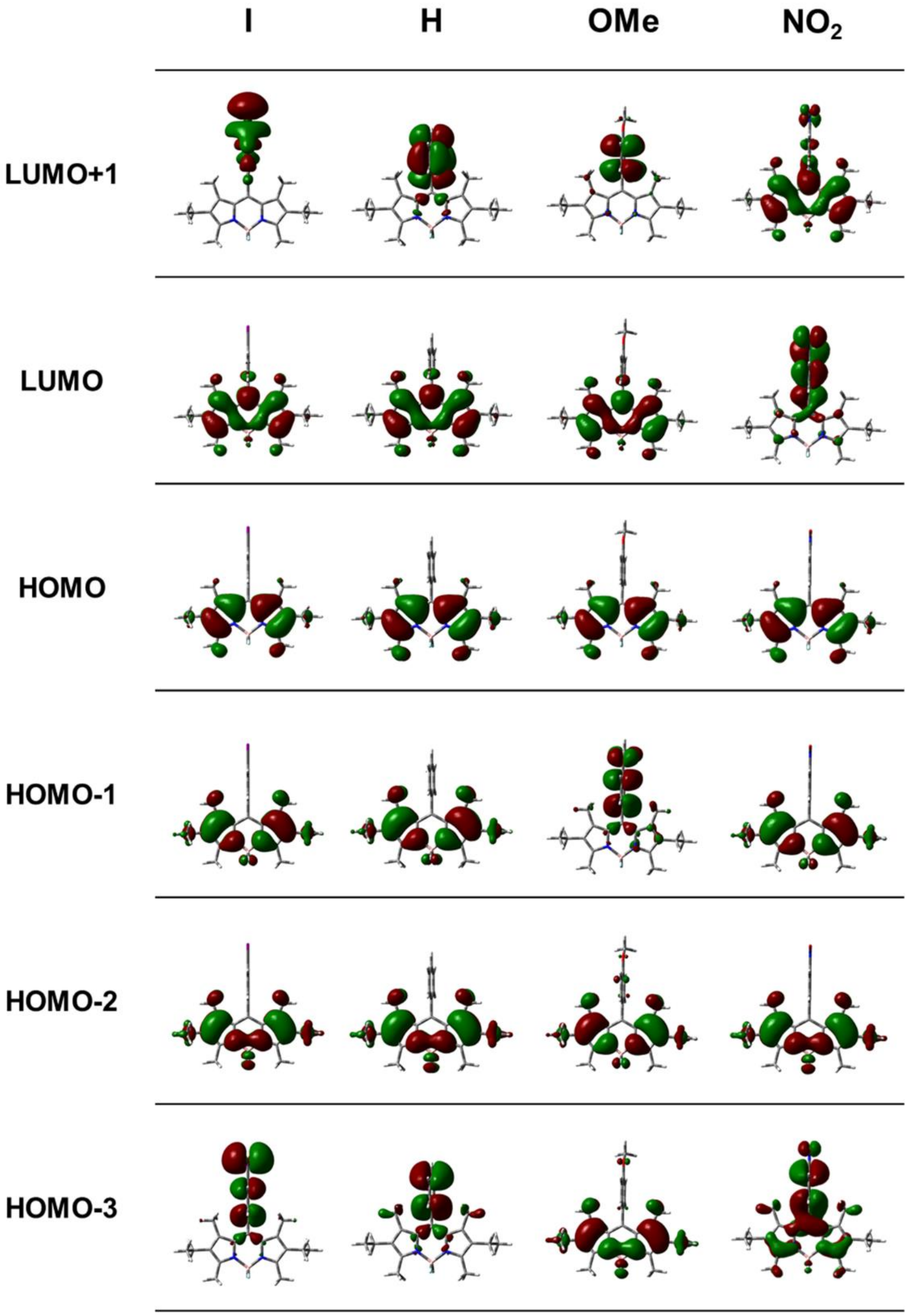

Figure S5. Frontier molecular orbitals of BODIPY **I, H, OMe**, and **NO2**.

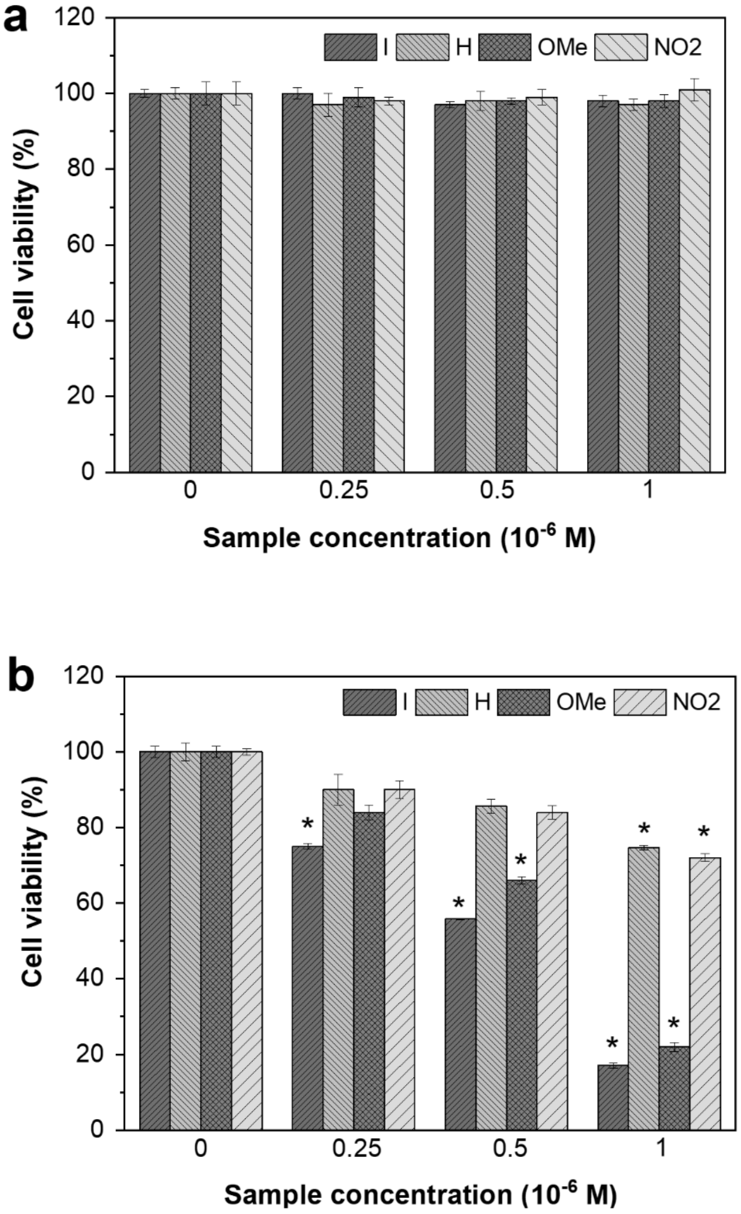

Figure S6. Cytotoxicity (a) and Phototoxicity (b) of BODIPY **I**, **H**, **OMe** and **NO2** in HeLa cells. Quantitative data for phototoxicity are expressed as a mean ± standard deviation (n=4). Statistical significance analyzed by Student's *t*-tests was considered as *$p<0.05$.

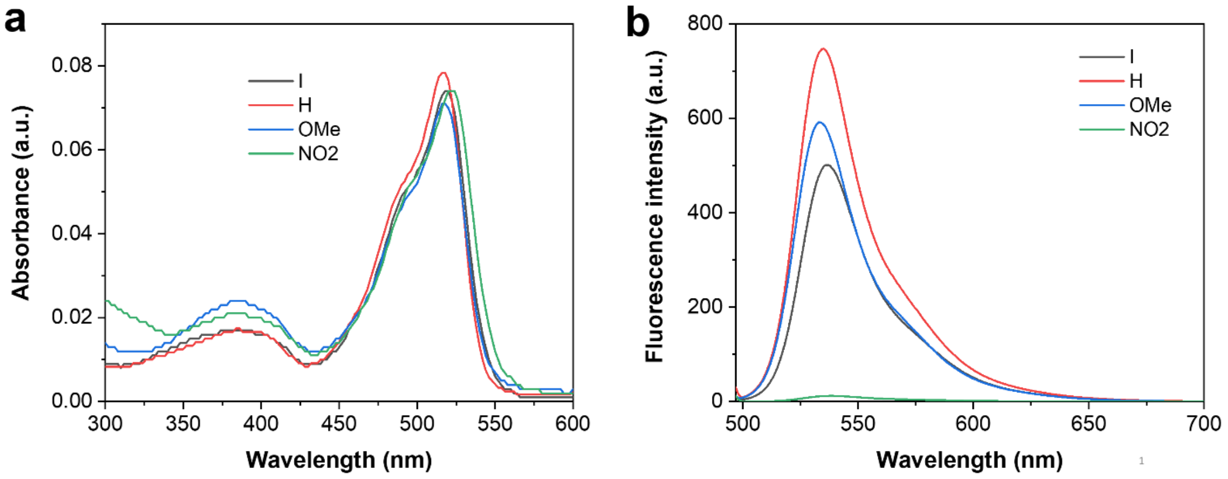

Figure S7. Absorption (a) and emission spectra (b) of BODIPY **I-NPs, H-NPs, OMe-NPs**, and **NO2-NPs** in aqueous solution at c= 5 µM. The solutions were excited at 490 nm.

Dye synthesis:

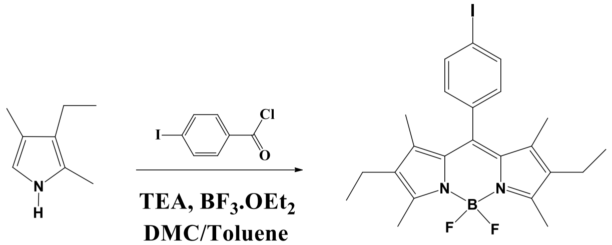

1a

BODIPY **1a** was synthesized according to our previous publication [1]. Briefly, 4-iodobenzoyl chloride (1.25 g, 4.69 mmol) and 3-ethyl-2, 4-dimethyl pyrrole (1.16 g, 9.42 mmol) were dissolved in 100 mL of dried CH_2Cl_2 at room temperature under Argon gas. After refluxing for 2h, the solvent was removed, and the residual mixture was dissolved with a mixture of toluene and CH_2Cl_2. Then TEA (2.6 mL) and $BF_3.Et_2O$ (2.6 mL) was then added to the mixture. After heating at 50^0C for 1.5 h, the solvent was evaporated, and the crude product was purified by column chromatography to afford **dye 1a** as an orange solid (0.763 g, 32% yield). ^{1}H-NMR (300 MHz, $CDCl_3$, δ, ppm): δ 7.85-7.83 (d, 2H), 7.06-7.04 (d, 2H), 2.53 (s, 6H), 2.32- 2.29 (q, 4H), 1.32 (s, 6H), 1.01- 0.96 (t, 6H).

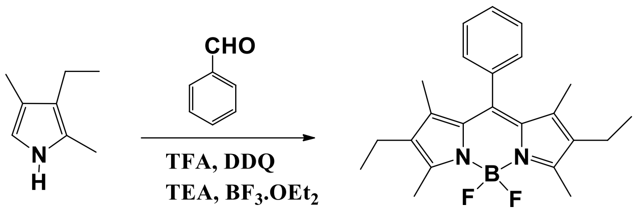

1b

Compound **1b** was synthesized as the same routine according to our previous publication [1]. In brief, 3-ethyl-2, 4-dimethyl pyrrole (0.84 g, 6.82 mmol), and benzaldehyde (0.36 g, 3.4 mmol) were dissolved in 100 mL of dried CH_2Cl_2 at room temperature under Argon gas in the presence of 2 drops of TFA. The DDQ (0.77 g, 3.4 mmol) was added to the mixture, followed by TEA (1.9 mL) and $BF_3.Et_2O$ (1.9 mL). BODIPY **1b** was afforded by column chromatography as a bright orange solid (0.26 g, 20% yield). ^{1}H-NMR (300 MHz, $CDCl_3$, δ, ppm): δ 7.49-7.46 (t, 3H), 7.3-7.26 (q, 2H), 2.53 (s, 6H), 2.33- 2.26 (q, 4H), 1.27 (s, 6H), 1.00- 0.95 (t, 6H).

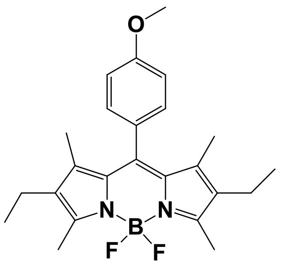

1c

Dye **1c** was afforded as the bright orange solid (210 mg, 25% yield). ^{1}H-NMR (300 MHz, $CDCl_3$, δ, ppm): δ 7.19-7.16 (d, dH), 7.02- 6.99 (d, 2H), 3.89 (s, 3H), 2.53 (s, 6H), 2.34- 2.27 (q, 4H), 1.33 (s, 6H), 1.01- 0.96 (t, 6H).

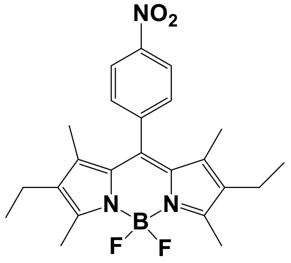

1d

BODIPY **1d** was afforded as a bright orange solid (240 mg, 23% yield). ^{1}H-NMR (300 MHz, $CDCl_3$, δ, ppm): δ 8.38-8.36 (d, 2H), 7.54-7.52 (d, 2H), 2.53 (s, 6H), 2.33- 2.26 (q, 4H), 1.25 (s, 6H), 1.00- 0.93 (t, 6H).

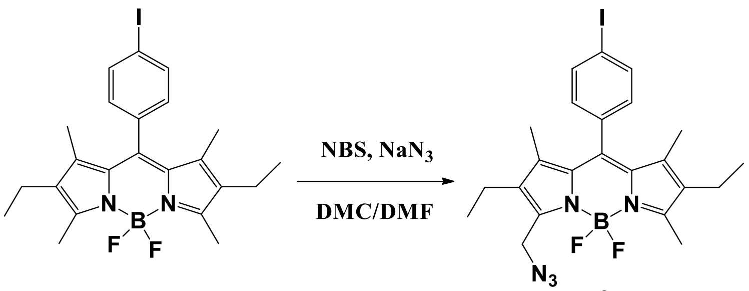

1a **2a**

BODIPY **2a** was synthesized using the same pathway according to the literature [2] with slightly modified. Briefly, dye **1a** (81 mg, 0.16 mmol) and NBS (34 mg, 0.19 mmol) were dissolved in the mixture of CH_2Cl_2 (16 mL), DMF (4 mL), following by adding of NaN_3 (208 mg, 3.2 mmol) and stirring overnight (for nucleophilic substitution); column chromatography on silica gel eluting with CH_2Cl_2/hexane to give dye **2a** as a bright orange solid (50 mg, 57%). ^{1}H-NMR (300 MHz, $CDCl_3$, δ, ppm): δ 7.88-7.85 (d, 2H), 7.08-7.05 (d, 2H), 4.58 (s, 2H), 2.57 (s, 3H), 2.39- 2.33 (q, 4H), 1.35 (s, 3H), 1.34 (s, 3H), 1.07- 0.99 (t, 6H).

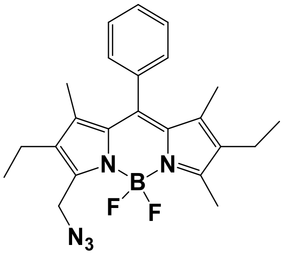

2b

Dye **2b** was collected as an orange solid (72 mg, 49% yield). ^{1}H-NMR (300 MHz, $CDCl_3$, δ, ppm): δ 7.52-7.50 (t, 3H), 7.30-7.28 (d, 2H), 4.59 (s, 2H), 2.58 (s, 3H), 2.39- 2.34 (q, 4H), 1.31 (s, 3H), 1.30 (s, 3H), 1.06- 0.98 (t, 6H).

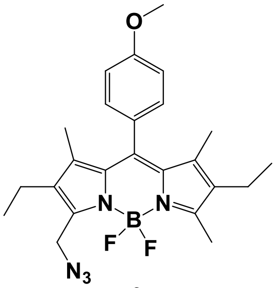

2c

Compound **2c** was prepared as an orange solid (70 mg, 64 % yield). ^{1}H-NMR (300 MHz, $CDCl_3$, δ, ppm): δ 7.20- 7.17 (d, 2H), 7.05-7.02 (d, 2H), 4.57 (s, 2H), 3.89 (s, 3H), 2.57 (s, 3H), 2.37- 2.31 (q, 4H), 1.36 (s, 3H), 1.35 (s, 3H), 1.06- 1.04 (t, 6H).

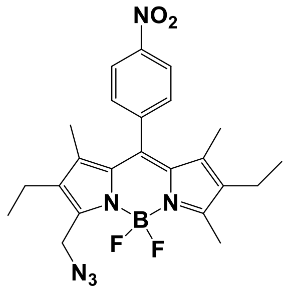

2d

Dye **2d** was afforded as an orange solid (80 mg, 73% yield). ^{1}H-NMR (300 MHz, $CDCl_3$, δ, ppm): δ 8.43- 8.39 (d, 2H), 7.58-7.55 (d, 2H), 4.59 (s, 2H), 2.60 (s, 3H), 2.39- 2.34 (q, 4H), 1.30 (s, 3H), 1.29 (s, 3H), 1.04- 0.96 (t, 6H).

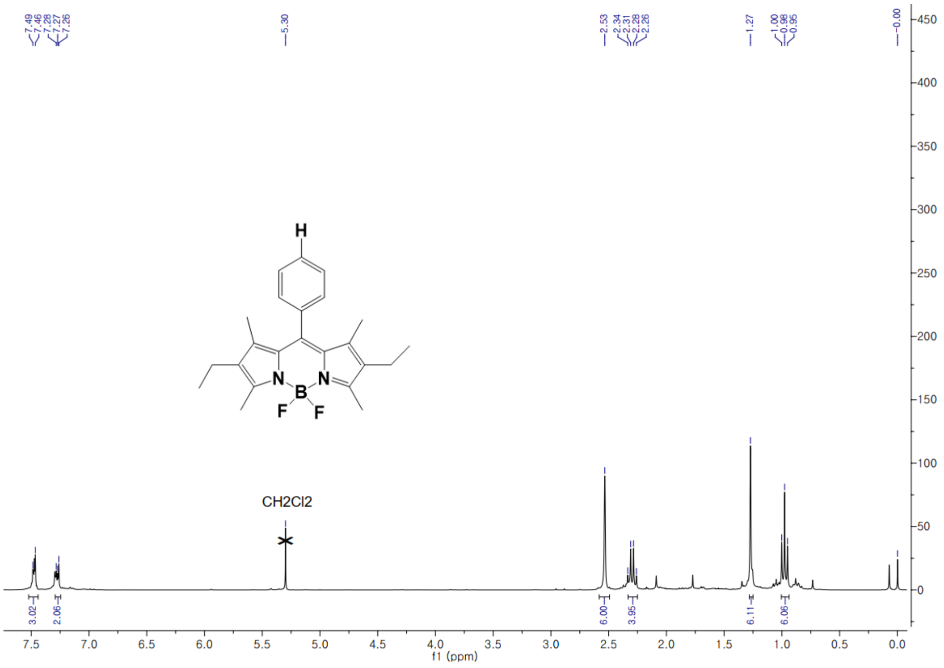

Figure S8. ^{1}H NMR spectrum of BODIPY **1b** in $CDCl_3$ at 300MHz

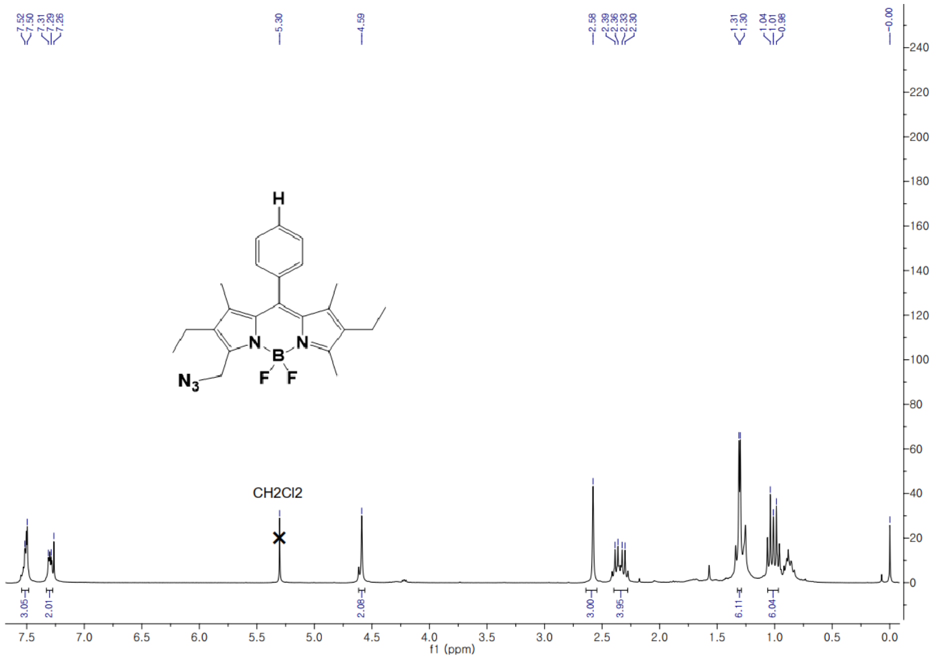

Figure S9. ^{1}H NMR spectrum of BODIPY **2b** in $CDCl_3$ at 300MHz

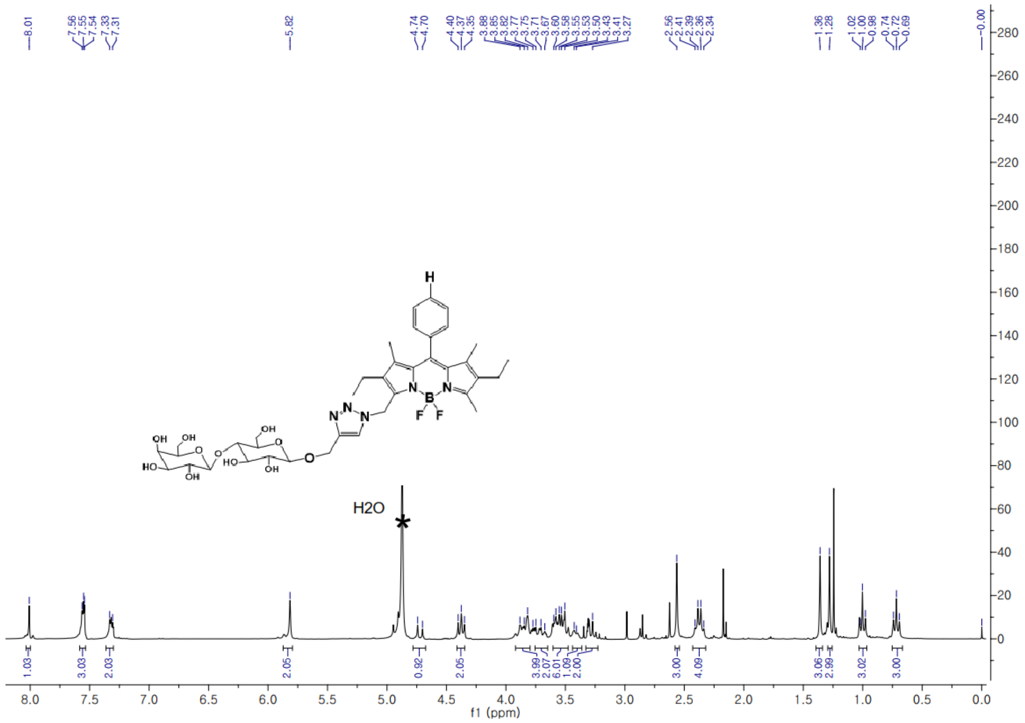

Figure S10. ^{1}H NMR spectrum of BODIPY **H** in CD_3OD at 300MHz

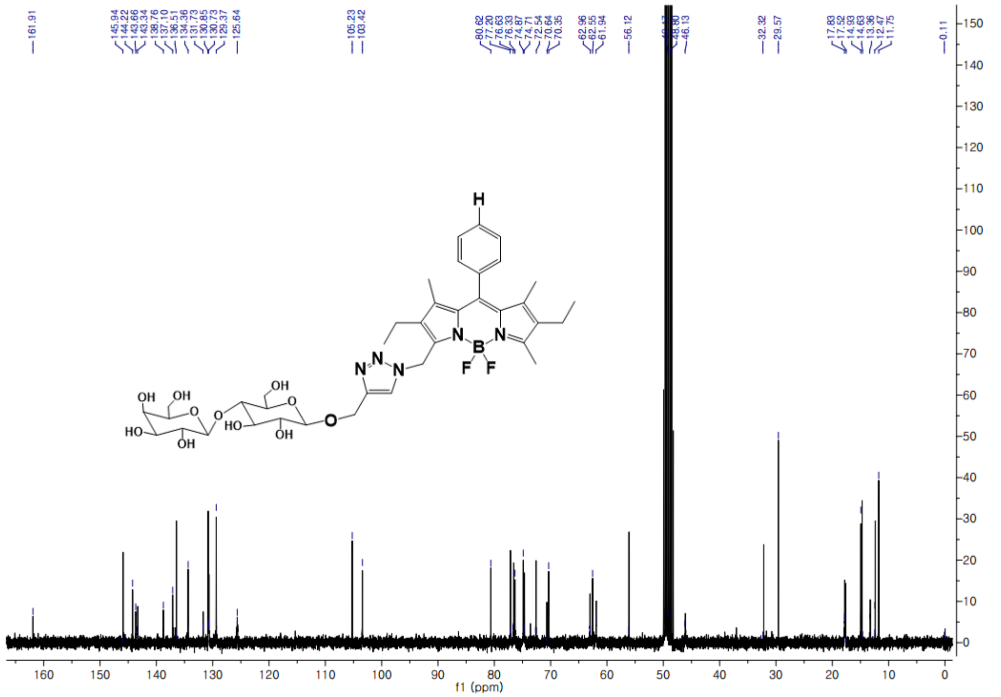

Figure S11. ^{13}C NMR spectrum of BODIPY **H** in CD_3OD at 75.4MHz

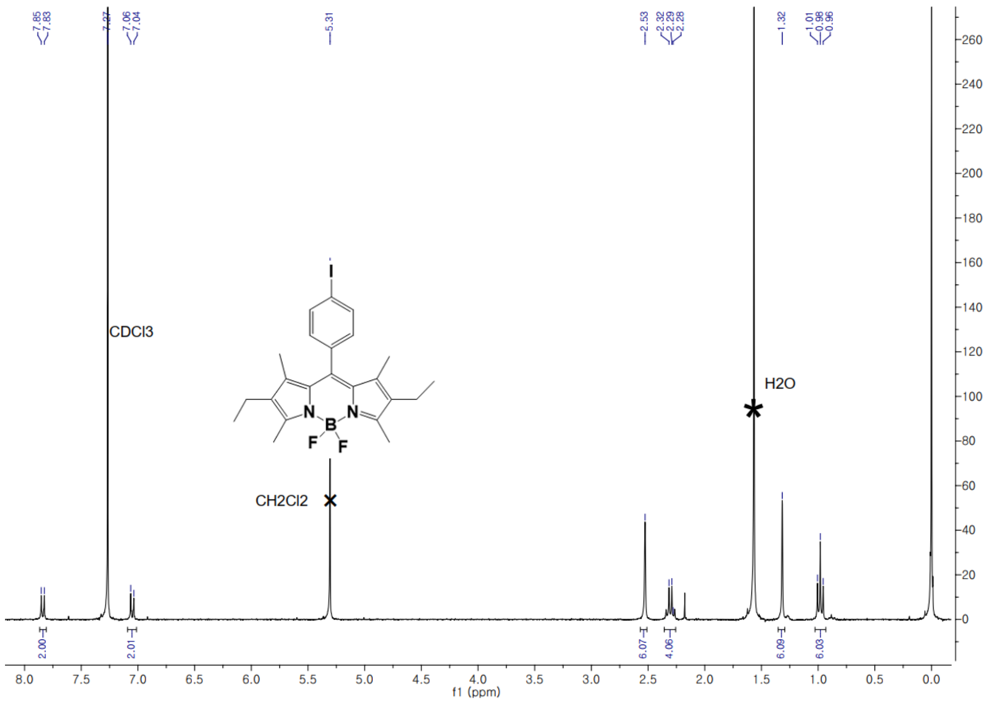

Figure S12. ^{1}H NMR spectrum of BODIPY **1a** in $CDCl_3$ at 300MHz

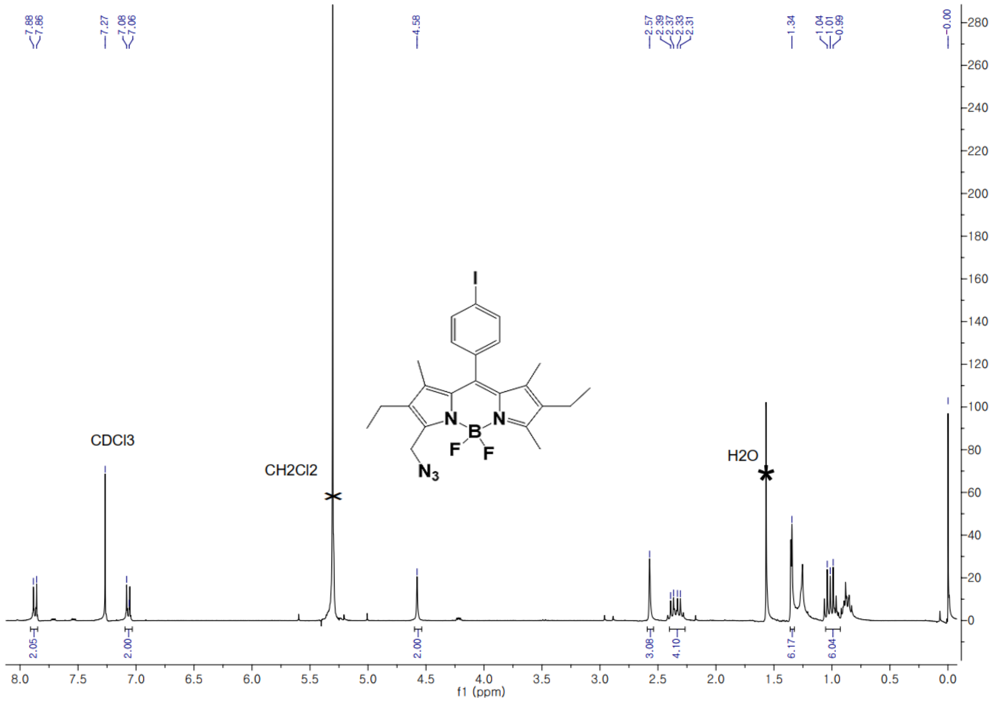

Figure S13. ^{1}H NMR spectrum of BODIPY **2a** in $CDCl_3$ at 300MHz

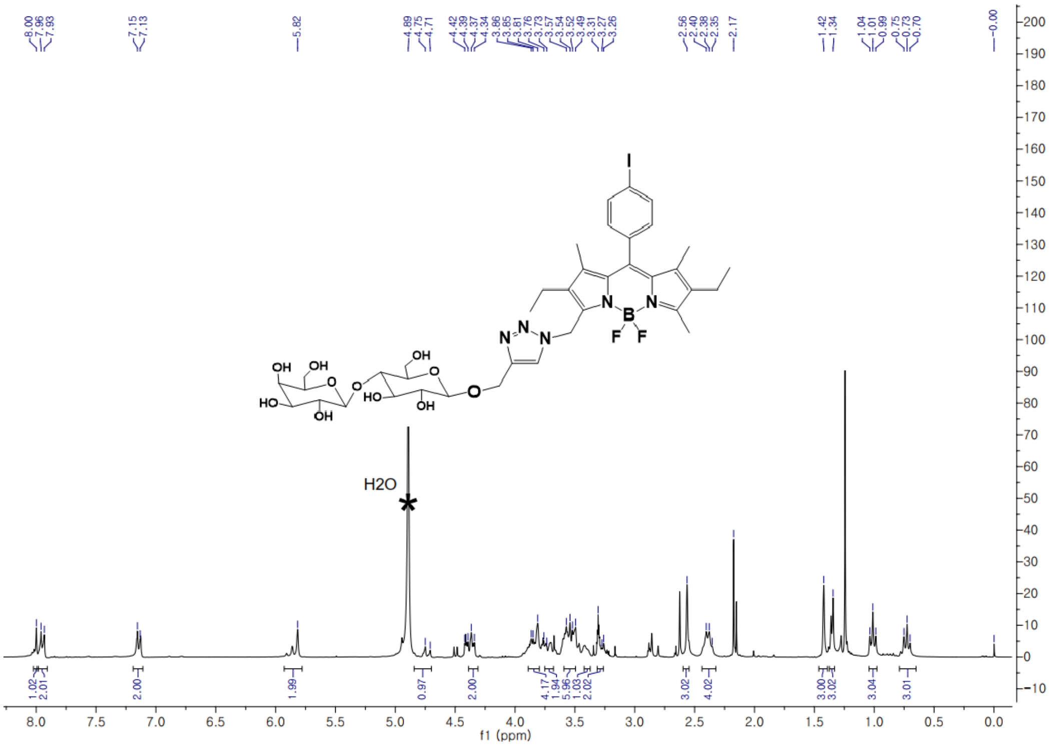

Figure S14. ^{1}H NMR spectrum of BODIPY **I** in CD_3OD at 300MHz

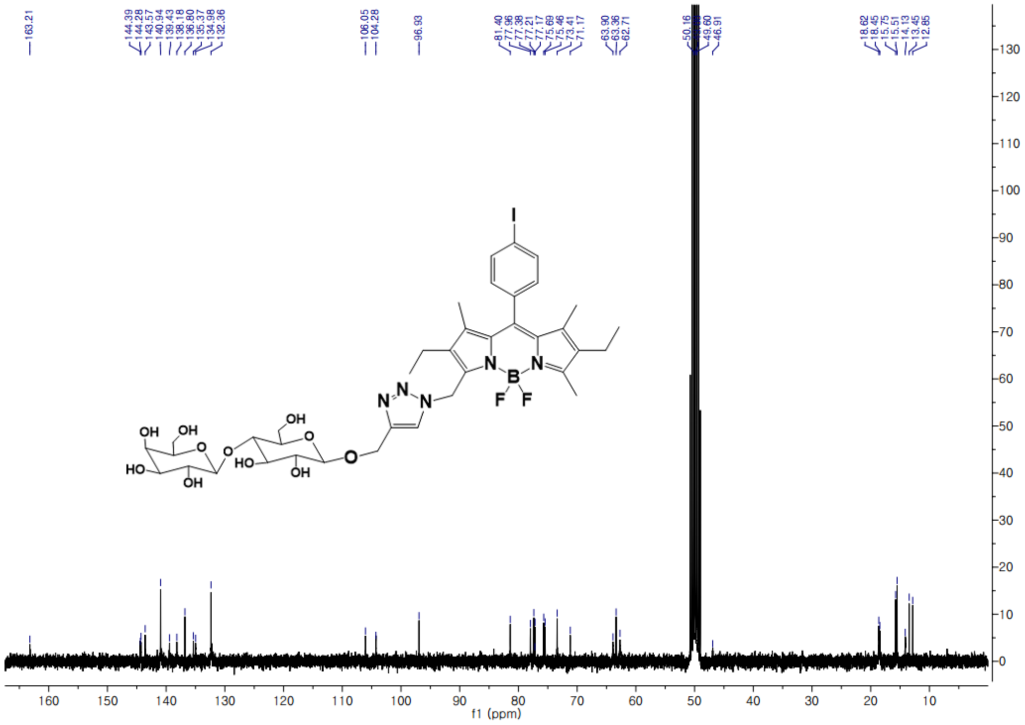

Figure S15. ^{13}C NMR spectrum of BODIPY **I** in CD_3OD at 75.4MHz

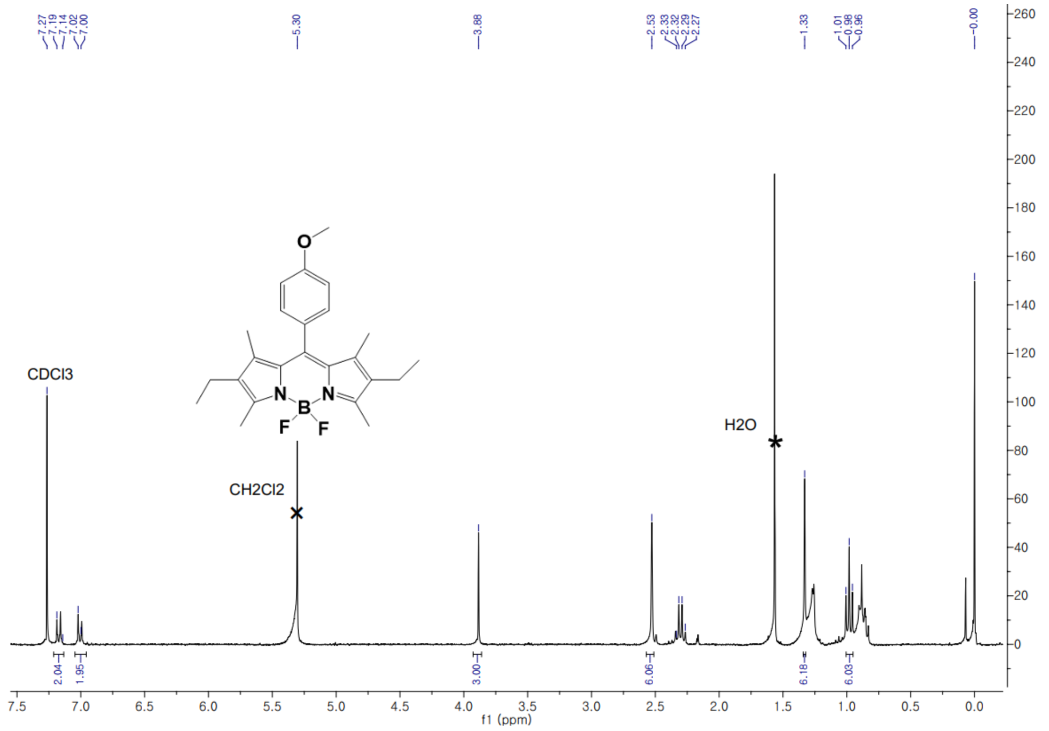

Figure S16. ^{1}H NMR spectrum of BODIPY **1c** in $CDCl_3$ at 300MHz

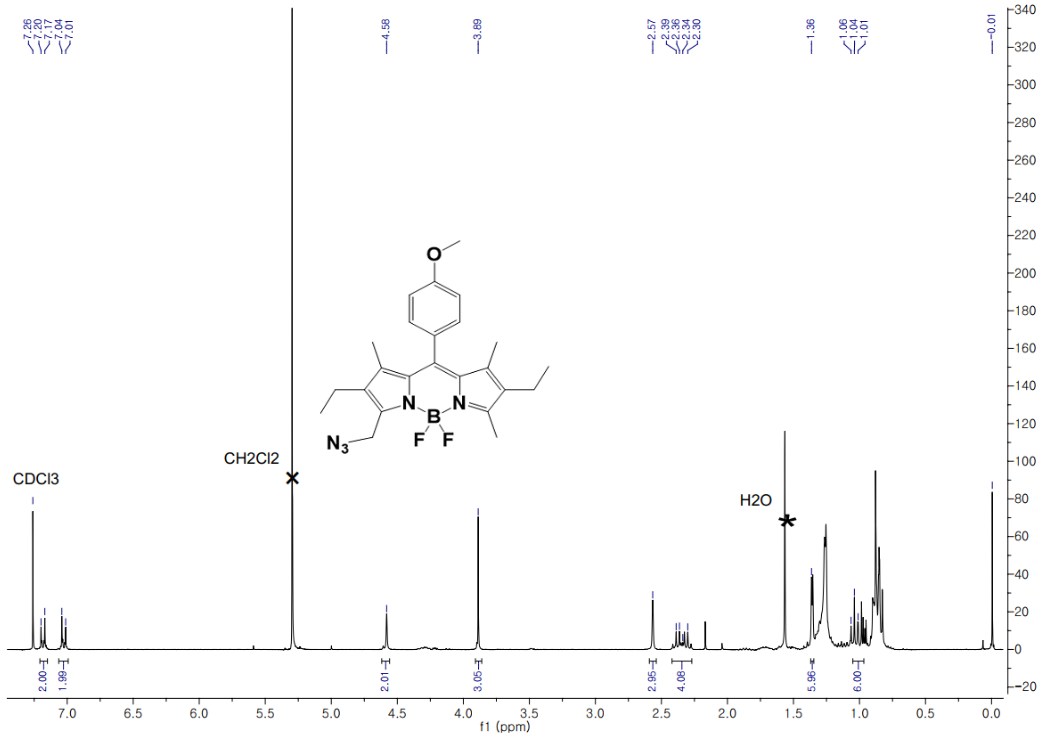

Figure S17. ^{1}H NMR spectrum of BODIPY **2c** in $CDCl_3$ at 300MHz

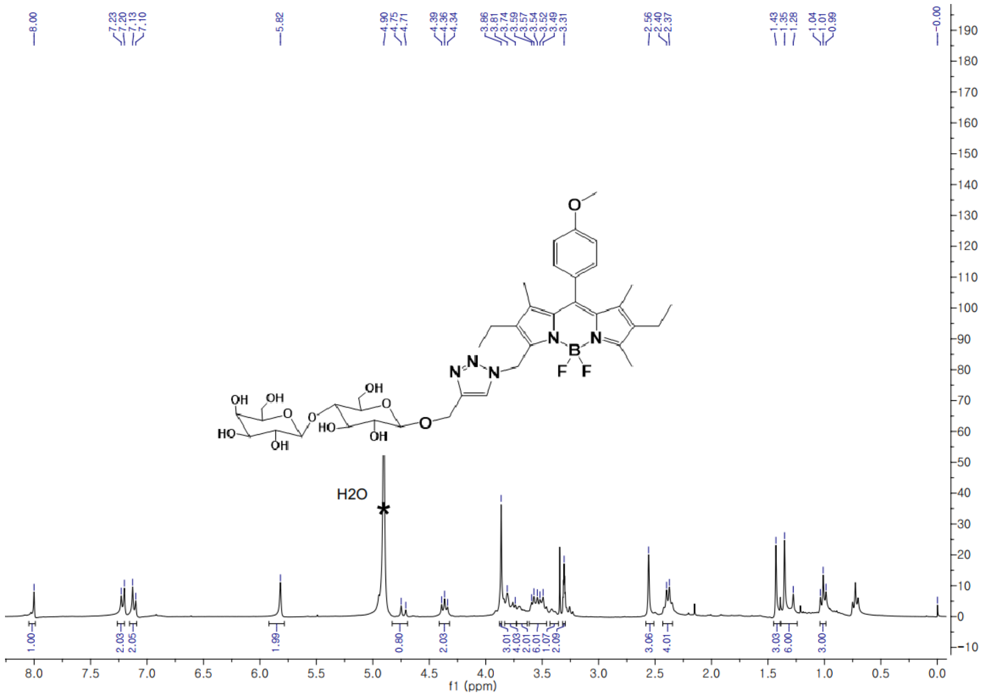

Figure S18. ^{1}H NMR spectrum of BODIPY **OMe** in CD_3OD at 300MHz

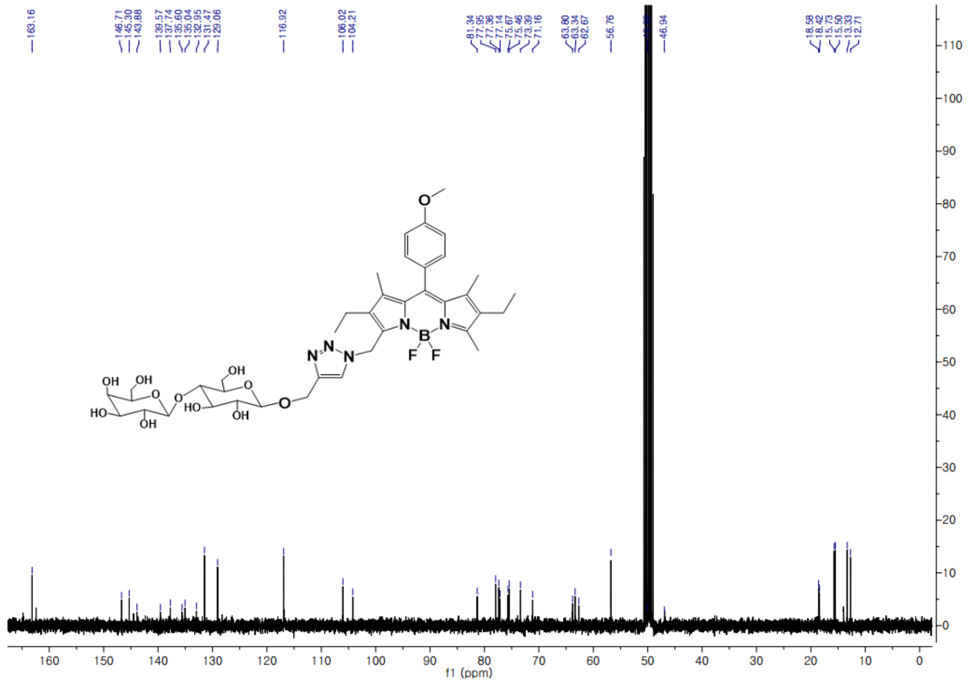

Figure S19. ^{13}C NMR spectrum of BODIPY **OMe** in CD_3OD at 75.4MHz

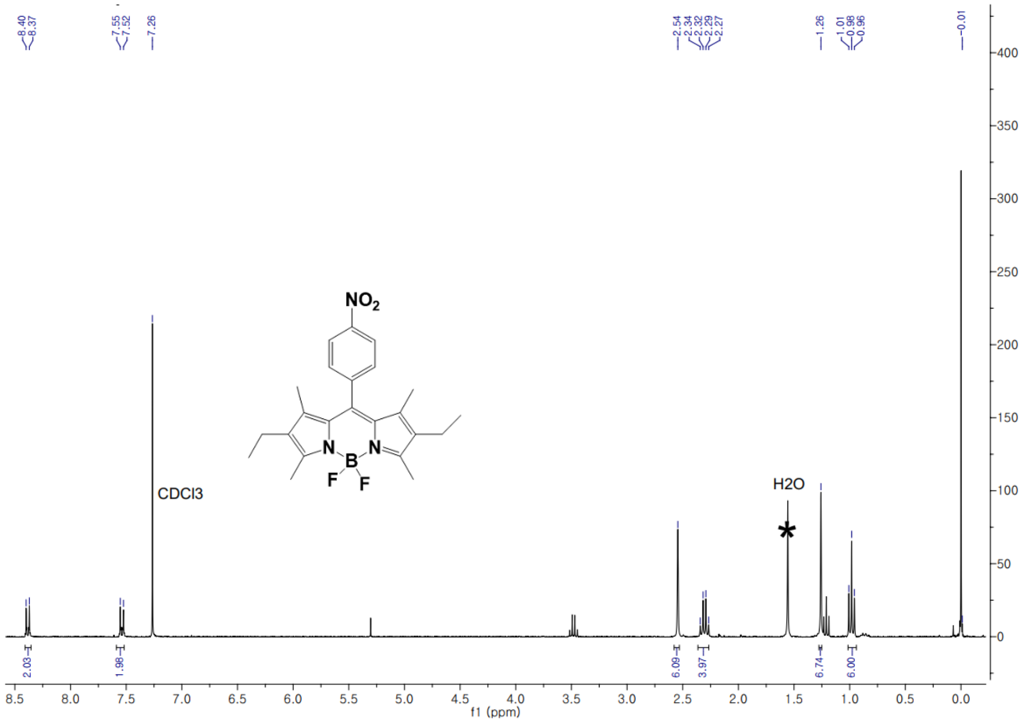

Figure S20. ¹H NMR spectrum of BODIPY **1d** in $CDCl_3$ at 300MHz

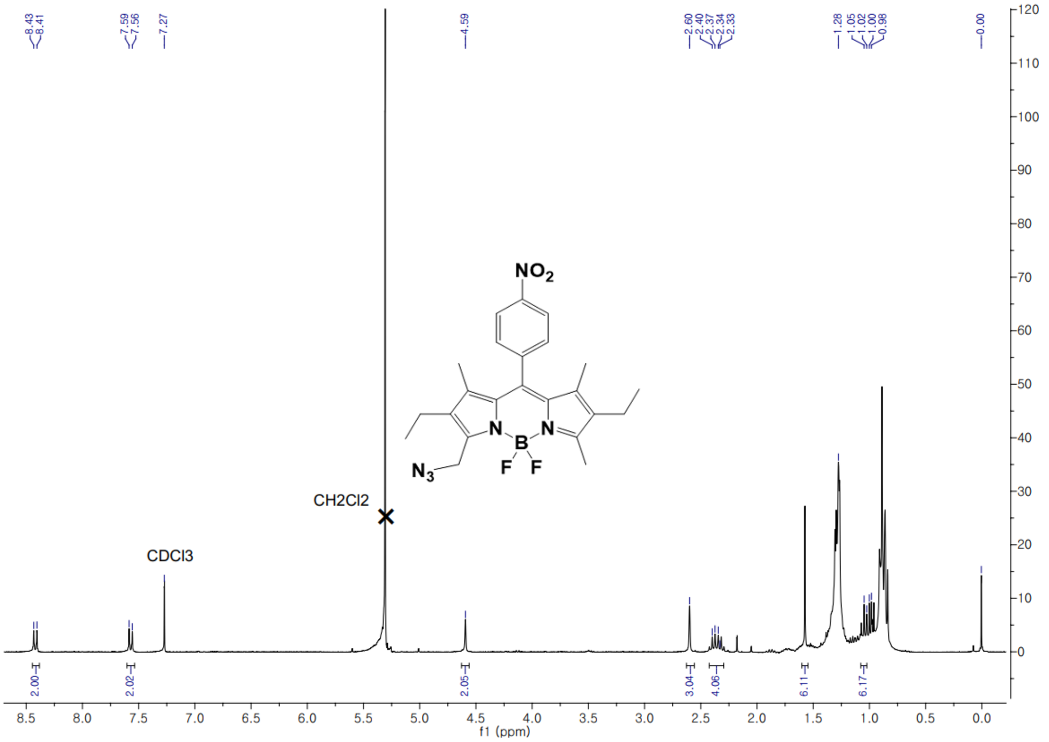

Figure S21. ¹H NMR spectrum of BODIPY **2d** in $CDCl_3$ at 300MHz

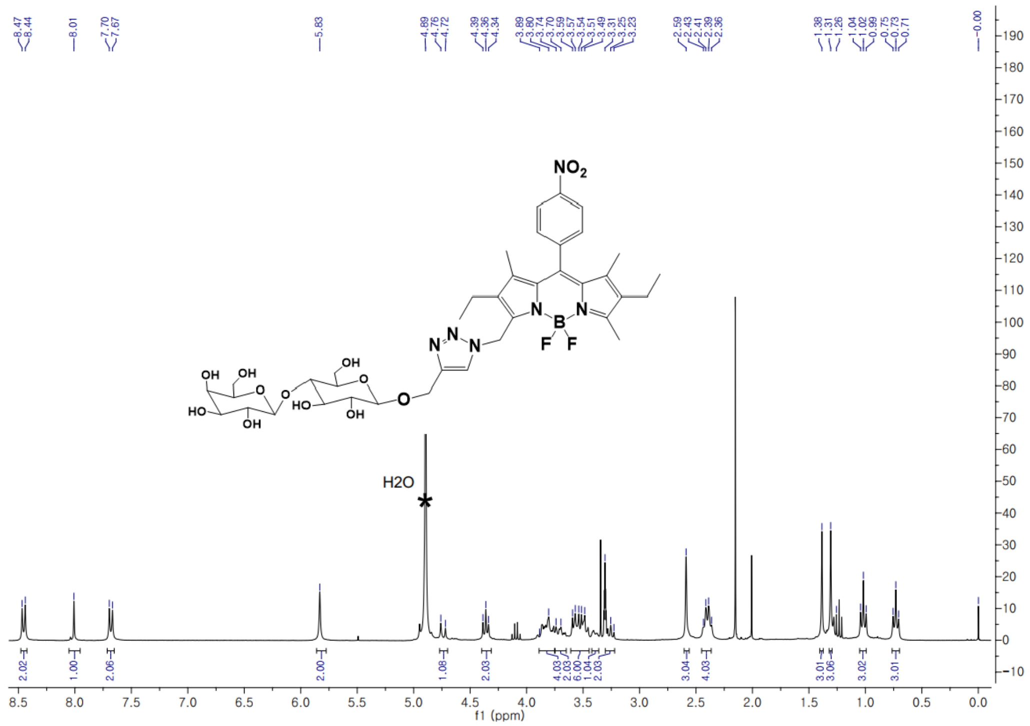

Figure S22. ^{1}H NMR spectrum of BODIPY **NO2** in CD_3OD at 300MHz

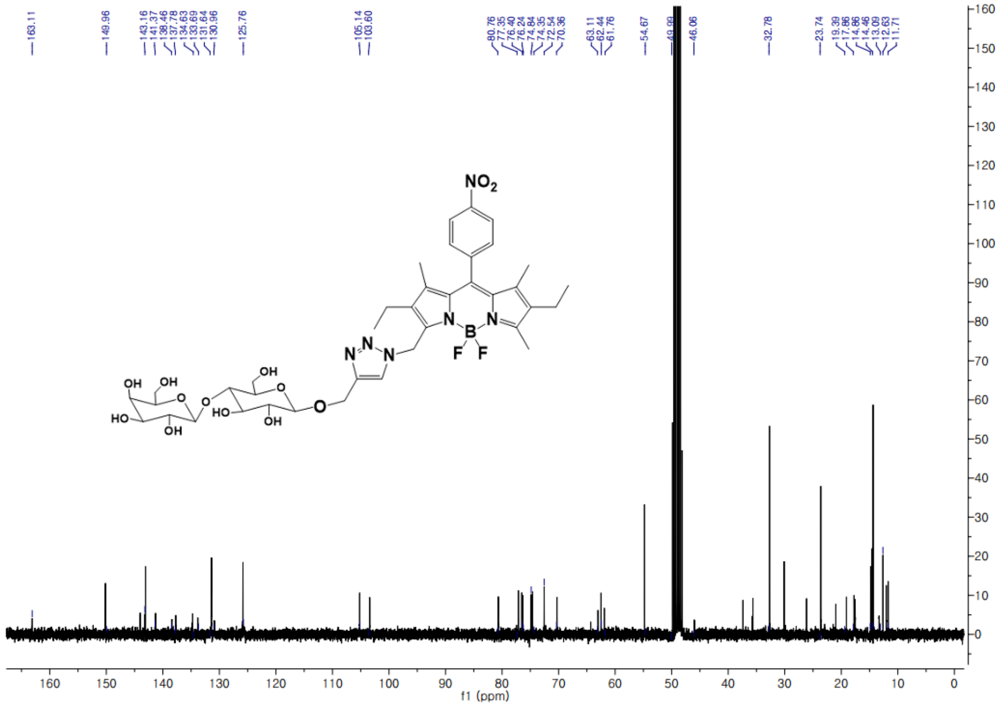

Figure S23. ^{13}C NMR spectrum of BODIPY **NO2** in CD_3OD at 75.4MHz

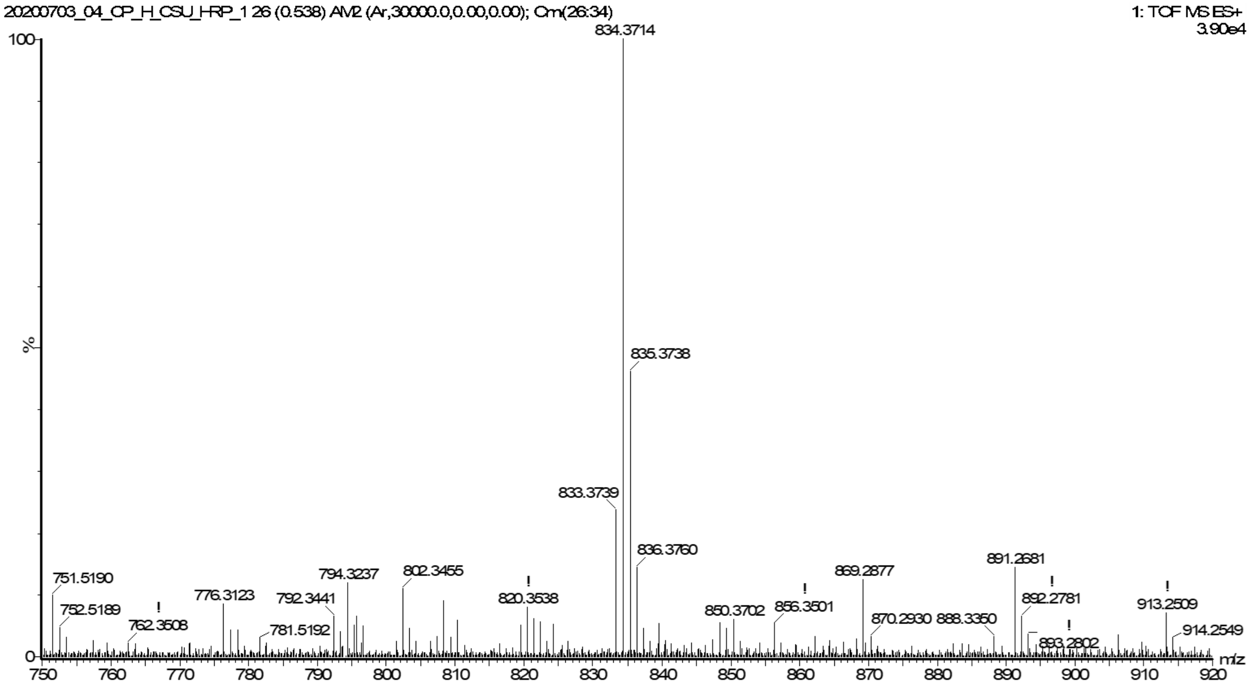

Figure S24. High-resolution mass spectrum (MS) of BODIPY **H**

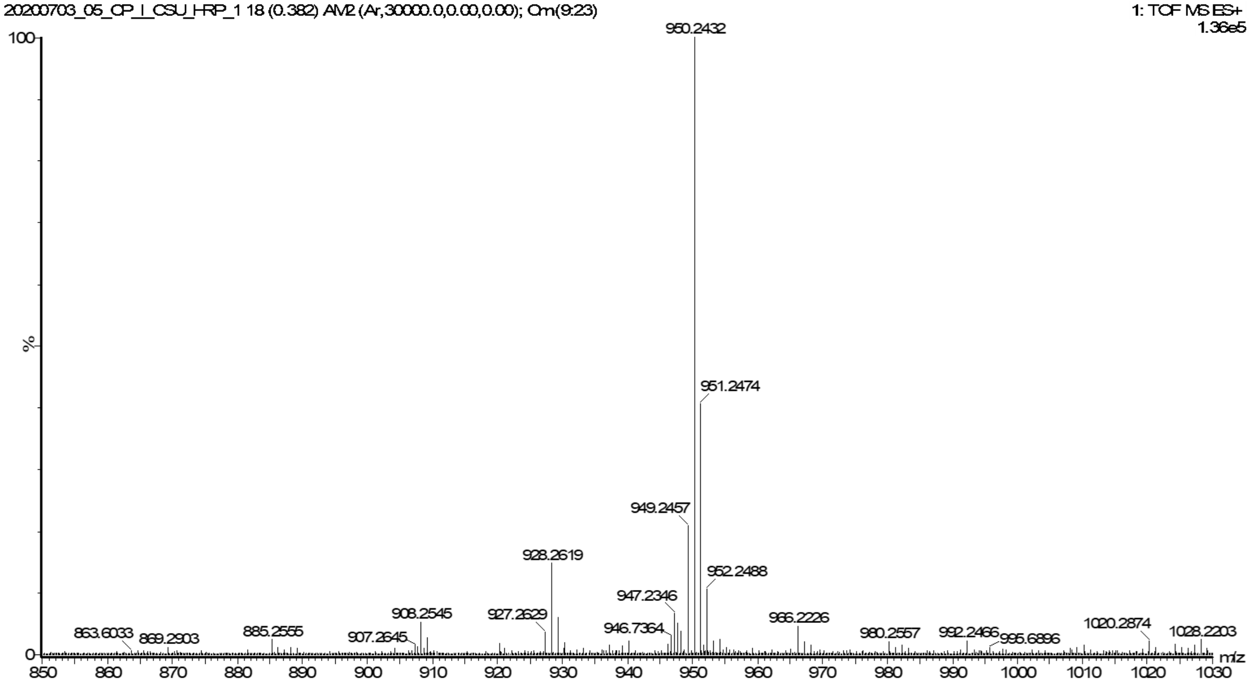

Figure S25. High-resolution mass spectrum (MS) of BODIPY **I**

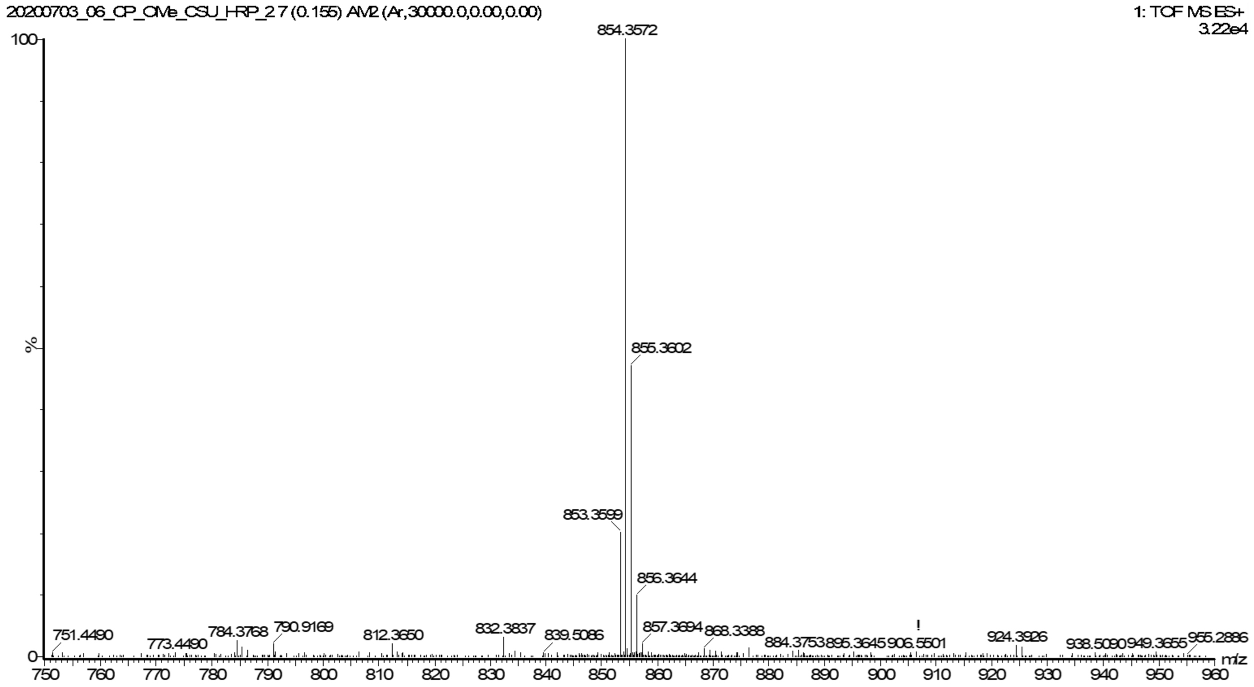

Figure S26. High-resolution mass spectrum (MS) of BODIPY **OMe**

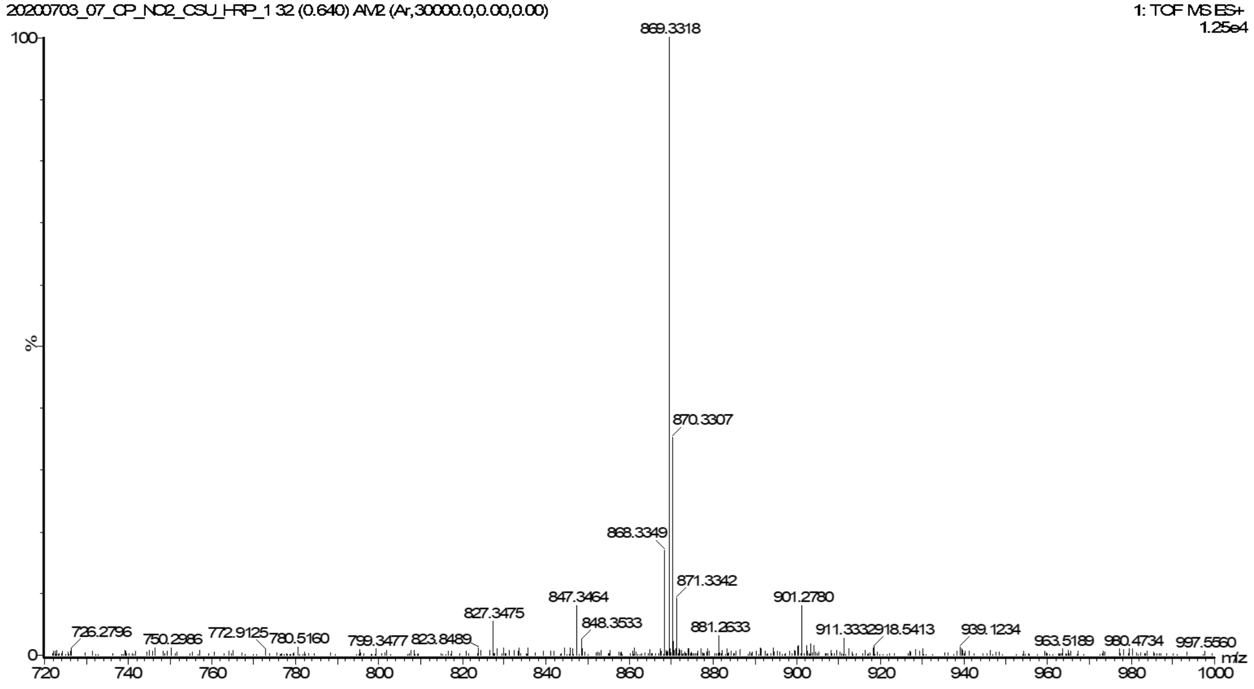

Figure S27. High-resolution mass spectrum (MS) of BODIPY **NO2**

Supporting References

1. Hoa Thi Bui, Duy Khuong Mai, Boram Kim, Kyong-Hoon Choi, Bong Joo Park, Ho-Joong Kim, and Sung Cho. *The Journal of Physical Chemistry B* **2019** *123* (26), 5601-5607
2. Gilles Ulrich, Raymond Ziessel, and Alexandre Haefele. *The Journal of Organic Chemistry* **2012** *77* (9), 4298-4311
